# Author Correction: Traps and transport resistance are the next frontiers for stable non-fullerene acceptor solar cells

**DOI:** 10.1038/s41467-022-32073-x

**Published:** 2022-08-02

**Authors:** Christopher Wöpke, Clemens Göhler, Maria Saladina, Xiaoyan Du, Li Nian, Christopher Greve, Chenhui Zhu, Kaila M. Yallum, Yvonne J. Hofstetter, David Becker-Koch, Ning Li, Thomas Heumüller, Ilya Milekhin, Dietrich R. T. Zahn, Christoph J. Brabec, Natalie Banerji, Yana Vaynzof, Eva M. Herzig, Roderick C. I. MacKenzie, Carsten Deibel

**Affiliations:** 1 Institut für Physik, Technische Universität Chemnitz, 09126 Chemnitz, Germany; 2 Institute of Materials for Electronics and Energy Technology (i-MEET), Friedrich-Alexander-Universität Erlangen-Nürnberg, 91054 Erlangen, Germany; 3 Helmholtz Institute Erlangen-Nürnberg for Renewable Energy (HI ERN), Immerwahrstrasse 2, 91058 Erlangen, Germany; 4 Guangdong Provincial Key Laboratory of Optical Information Materials and Technology, Institute of Electronic Paper Displays, South China Academy of Advanced Optoelectronics, South China Normal University, Guangzhou, 510006 P. R. China; 5 Physikalisches Institut, Dynamik und Strukturbildung - Herzig Group, Universität Bayreuth, Universitätsstr. 30, 95447 Bayreuth, Germany; 6 Advanced Light Source, Lawrence Berkeley National Laboratory, Berkeley, CA 94720 USA; 7 Department of Chemistry and Biochemistry, University of Bern, 3012 Bern, Switzerland; 8 Integrated Center for Applied Photophysics and Photonic Materials, Technische Universität Dresden, 01062 Dresden, Germany; 9 Center for Advancing Electronics Dresden, Technische Universität Dresden, 01062 Dresden, Germany; 10 State Key Laboratory of Luminescent Materials and Devices, Institute of Polymer Optoelectronic Materials and Devices, School of Materials Science and Engineering, South China University of Technology, 381 Wushan Road, 510640 Guangzhou, China; 11 Department of Engineering, Durham University, Lower Mount Joy, South Road, Durham, DH1 3LE UK

**Keywords:** Photonic devices, Polymers, Solar cells, Solar cells, Electronic and spintronic devices

Correction to: *Nature Communications* 10.1038/s41467-022-31326-z, published online 01 July 2022

The original version of this Article contained an error in Fig. 2, in which three data sets (represented by circles, squares and triangles, respectively) were shifted along the x-axis by one data point, leading to an incorrect representation of the “Exp. suns-*V*_oc_” data (the symbols) in Fig. 2a and 2b. The correct version of Fig. 2 is:
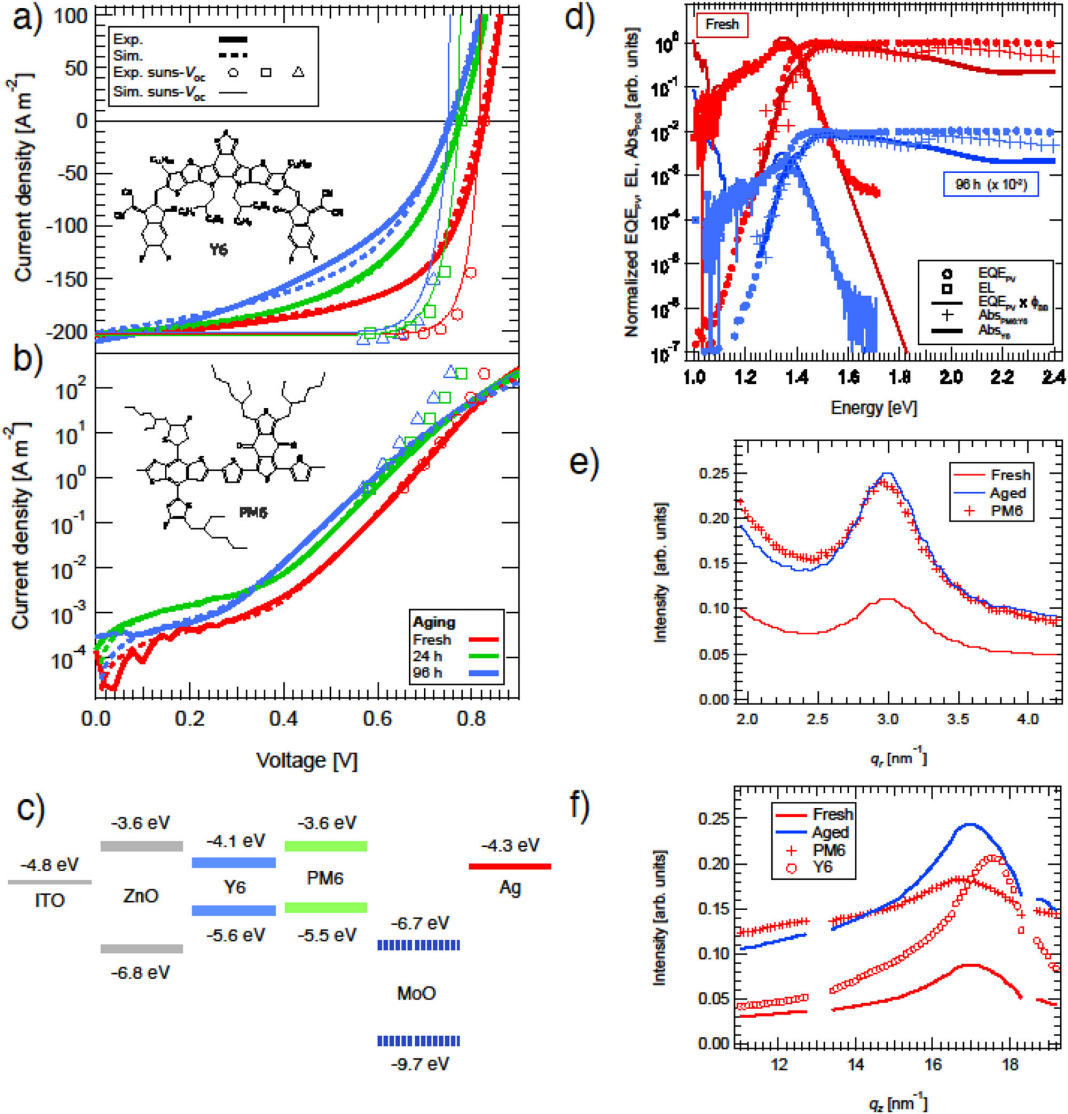


which replaces the previous incorrect version:
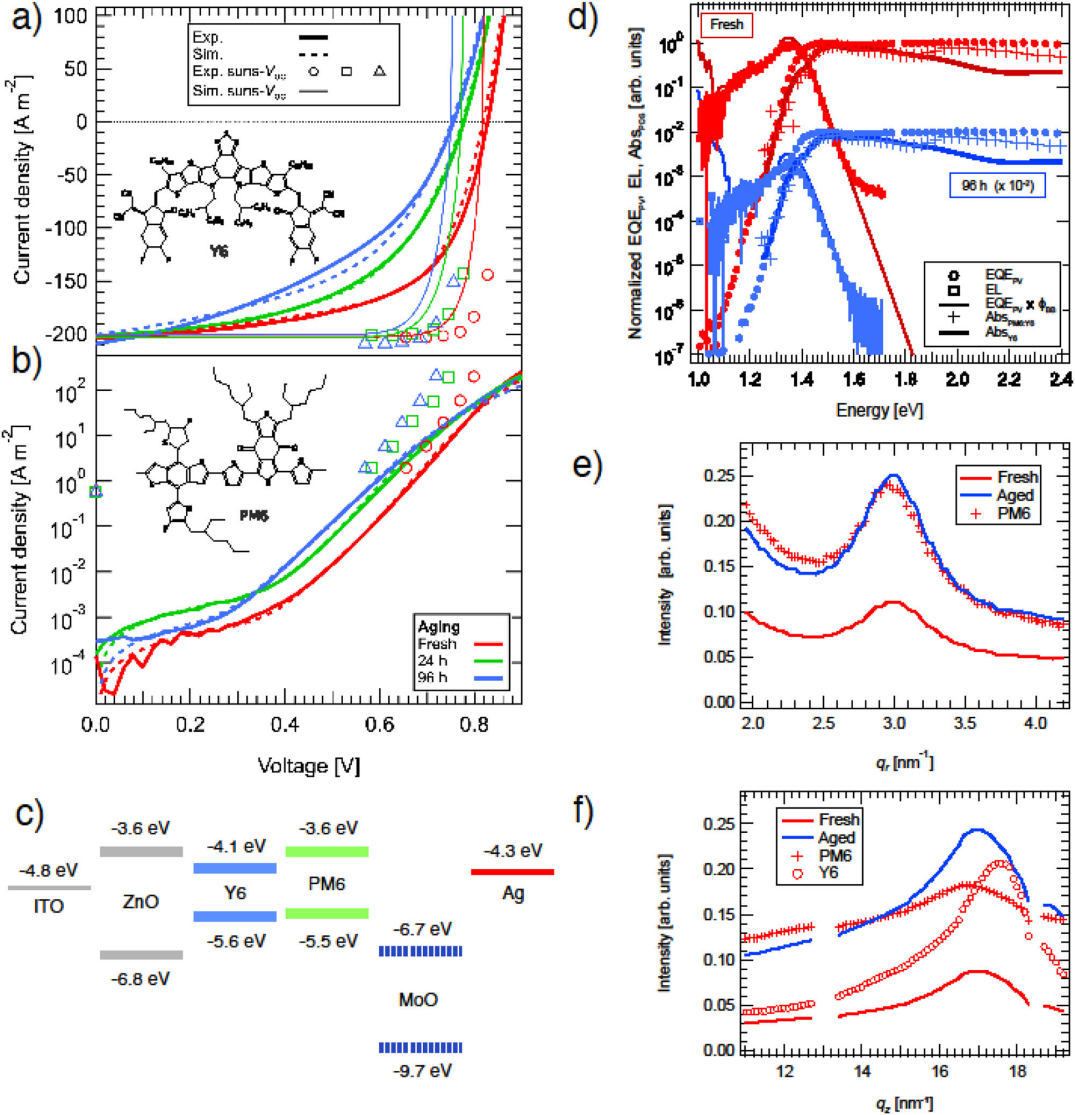


This has been corrected in both the PDF and HTML versions of the Article.

