# Supplementary material for: Traps and transport resistance are the next frontiers for stable non-fullerene acceptor solar cells
Source: Nat Commun. 2022 Jul 1;13:3786. doi: 10.1038/s41467-022-31326-z (PMC9249898; doi:10.1038/s41467-022-31326-z)
Supplement: Supplementary file 1 — Supplementary Information [file 41467_2022_31326_MOESM1_ESM.pdf]

Supplementary Information for
Traps and transport resistance are the next frontiers for stable non-fullerene acceptor solar cells

Christopher Wöpke,[1] Clemens Göhler,[1] Maria Saladina,[1] Xiaoyan Du,[2,3] Li Nian,[2,4] Christopher Greve,[5] Chenhui Zhu,[6] Kaila M. Yallum,[7] Yvonne J. Hofstetter,[8,9] David Becker-Koch,[8,9] Ning Li,[2,3,10] Thomas Heumüller,[2,3] Ilya Milekhin,[1] Dietrich R. T. Zahn,[1] Christoph J. Brabec,[2,3] Natalie Banerji,[7] Yana Vaynzof,[8,9] Eva M. Herzig,[5] Roderick C. I. MacKenzie,[11] and Carsten Deibel[1,*]

[1]*Institut für Physik, Technische Universität Chemnitz, 09126 Chemnitz, Germany*
[2]*Institute of Materials for Electronics and Energy Technology (i-MEET), Friedrich-Alexander-Universität Erlangen-Nürnberg, 91054 Erlangen, Germany*
[3]*Helmholtz Institute Erlangen-Nürnberg for Renewable Energy (HI ERN), Immerwahrstrasse 2, 91058 Erlangen, Germany*
[4]*Guangdong Provincial Key Laboratory of Optical Information Materials and Technology, Institute of Electronic Paper Displays, South China Academy of Advanced Optoelectronics, South China Normal University, Guangzhou, 510006 P. R. China*
[5]*Physikalisches Institut, Dynamik und Strukturbildung - Herzig Group, Universität Bayreuth, Universitätsstr. 30, 95447 Bayreuth, Germany*
[6]*Advanced Light Source, Lawrence Berkeley National Laboratory, Berkeley, California 94720, United States*
[7]*Department of Chemistry and Biochemistry, University of Bern, 3012 Bern, Switzerland*
[8]*Integrated Center for Applied Photophysics and Photonic Materials, Technische Universität Dresden, 01062 Dresden, Germany*
[9]*Center for Advancing Electronics Dresden, Technische Universität Dresden, 01062 Dresden, Germany*
[10]*State Key Laboratory of Luminescent Materials and Devices, Institute of Polymer Optoelectronic Materials and Devices, School of Materials Science and Engineering, South China University of Technology, 381 Wushan Road, 510640 Guangzhou, China*
[11]*Department of Engineering, Durham University, Lower Mount Joy, South Road, Durham, DH1 3LE, UK*

Contents

I. Variable angle spectroscopy ellipsometry (VASE)
II. Transient absorption (TA) spectroscopy
III. EQE_{PV} sub-bandgap energetics
IV. Conductivity
V. Capacitance–voltage measurements
VI. Intensity-modulated spectroscopy
VII. Grazing Incidence Wide Angle X-Ray Scattering (GIWAXS)
VIII. Ultraviolet photoemission spectroscopy (UPS)
IX. Details of the device simulation
References

* email: deibel@physik.tu-chemnitz.de

Supplementary Note I. Variable angle spectroscopy ellipsometry (VASE)

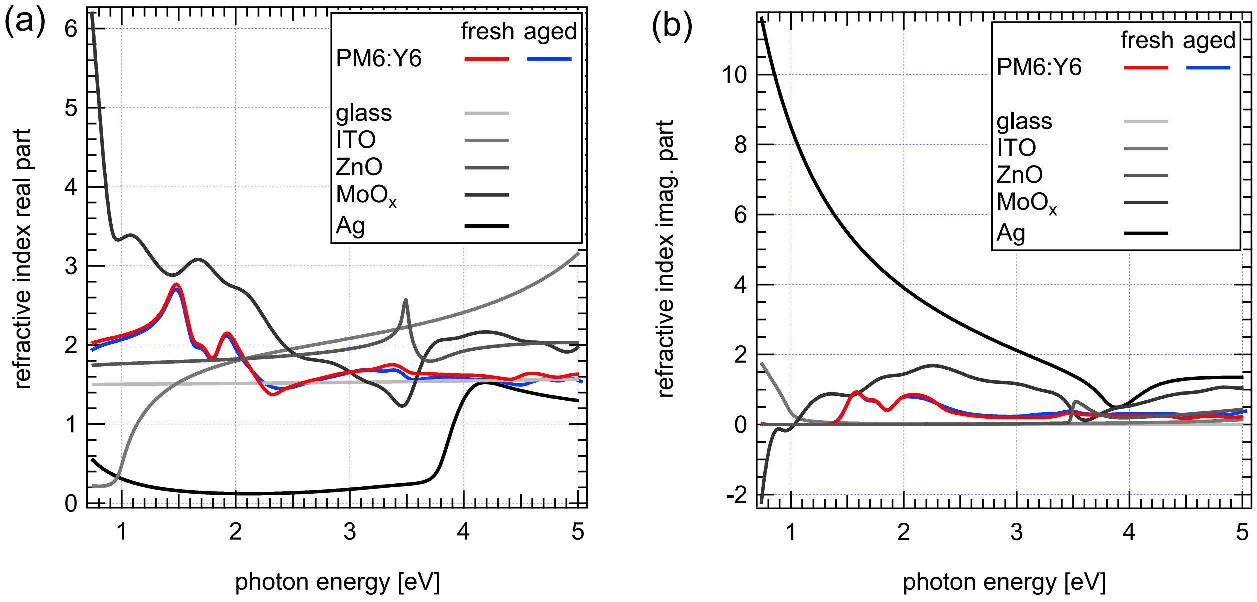

Supplementary Figure 1. VASE measurements on fresh and aged samples. Optical constants (a) n and (b) k measured with VASE for every layer of the solar cell stack as well as for a complete device, fresh and aged.

At first, the layer thickness of ITO and ZnO was determined from the spectra recorded on the ITO/ZnO bilayer structure on the glass substrate. The optical constants of the glass, ITO, and ZnO were used from Woollam's CompleteEASE database and the thickness of ITO and ZnO were the fit parameters. Once a reasonable fit of experimental data and optical model was obtained (multi-sample analysis, MSA), the optical constants of ITO and ZnO were tuned to best fit the experimental data. The optical constants and the thickness of ITO and ZnO were kept fixed in the proceeding samples.

After three samples with the varying thickness of PM6:Y6 mixture deposited on glass substrate/ITO/ZnO. The spectra from these samples were modeled using the MSA method. For this, the optical constants of the PM6:Y6 mixture were the common fit parameters, whereas the thickness was an independent fit parameter for each spectrum. The optical dispersion function of the PM6:Y6 mixture was modeled with a Kramers-Kronig constrained fixed 22 node B-Spline function.[1] For the next set of samples, the optical constant of the PM6:Y6 mixture was kept fixed. To probe the influence of aging on the optical properties of the PM6:Y6 mixture three samples identical to the above-discussed set of samples were aged for 100 h and were measured similarly.

A complete solar cell comprising of glass/ITO/ZnO/ PM6:Y6/MoO_x/Ag was prepared with the varying thickness of MoO_x layer passivated with the Ag layer of known thickness. Once again, the MSA approach was used to determine the optical constants and thickness of the MoO_x layer, while the optical constant of the Ag was used from the CompleteEASE database and the thickness of the Ag was kept fixed to 2.0 nm. The thickness of the Ag layer was determined from the quartz microbalance measurement during the deposition. Similar to the PM6:Y6 mixture, the dispersion relation of the MoO_x layer was modeled with a 22 nodes B-Spline function following the Kramer-Kronig constraint. The nodes were matched to the optical constants of MoO_3[2] as an estimate before fitting to improve the chance of finding a correct solution.

Supplementary Note II. Transient absorption (TA) spectroscopy

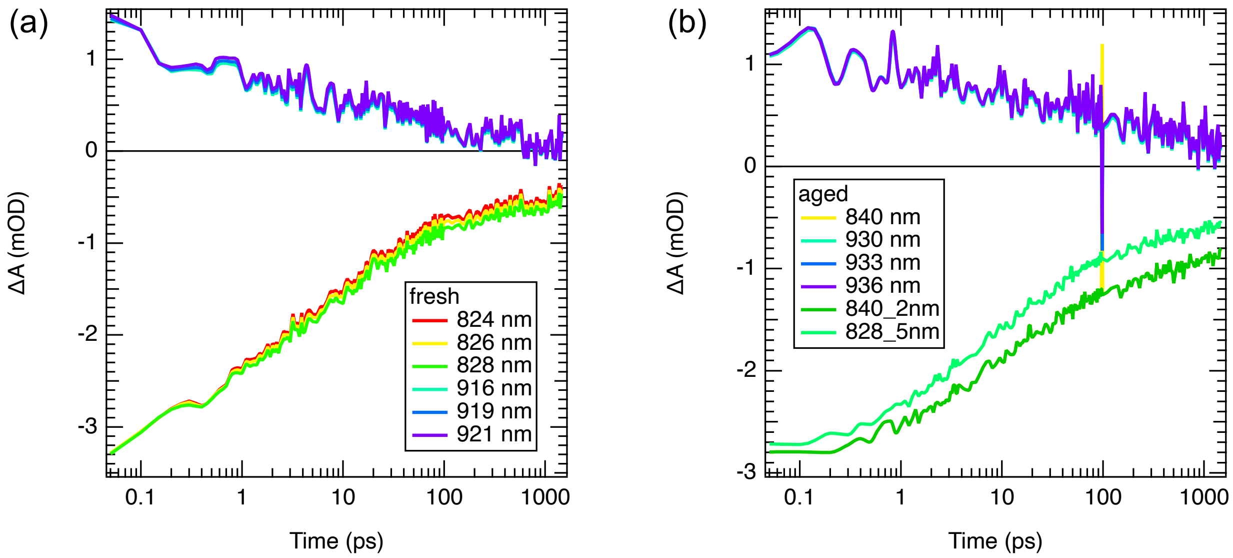

Supplementary Figure 2. Transient absorption spectroscopy of (a) fresh and (b) aged PM6:Y6 thin films.

Supplementary Note III. $\mathrm{EQE_{PV}}$ sub-bandgap energetics

The low absorbing part of $\mathrm{EQE_{PV}}$ can be approximated by an exponential distribution of states; the slope is identified with the corresponding Urbach energy E_U

$$\mathrm{EQE_{PV}} \propto \exp\left(\frac{E}{E_\mathrm{U}}\right) \tag{1}$$

As is shown in Fig. 3, E_U varies slightly between both cell thicknesses, yet remains unaffected by aging.

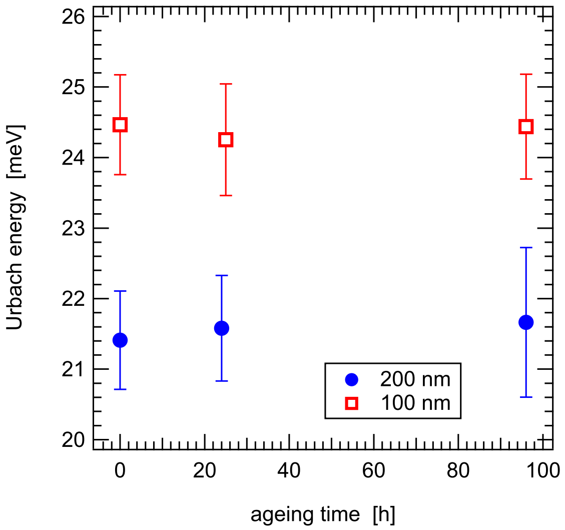

Supplementary Figure 3. Urbach energies of the sub-bandgap tail state distributions. Thicker cells exhibit a slightly lower Urbach energy, which in both cases remains unaffected by aging. Error bars indicate one standard deviation given by fitting Eq. (1).

Influence of radiative efficiency on V_oc. If the electroluminescence quantum yield $\mathrm{QY_{EL}}$ follows a distinct temperature dependence,[3] we would expect a substantial influence on the quasi-Fermi-level splitting. From the Shockley equation with ideality factor $n \neq 1$, we find

$$\mathrm{QY} = \mathrm{QY}^0 J_\mathrm{inj}^{n-1} \exp\left(\frac{-\Delta E}{kT}\right) \tag{2}$$

$$J_\mathrm{inj} = \frac{J_0^\mathrm{rad}}{\mathrm{QY}} \exp\left(\frac{qV}{kT}\right) \tag{3}$$

$$J(V) = \left(\frac{J_0^\mathrm{rad}}{\mathrm{QY}^0}\right)^{1/n} \exp\left(\frac{\Delta E}{nkT}\right)\left(\exp\left(\frac{qV}{nkT}\right) - 1\right) - J_\mathrm{sc} \tag{4}$$

Under open-circuit conditions ($J(V) = 0$), the energy barrier ΔE works as a constant difference to the radiative limit given by J_0^rad

$$qV_\mathrm{oc} \approx nkT \ln\left(J_\mathrm{sc}\right) - nkT \ln\left(\frac{J_0^\mathrm{rad}}{\mathrm{QY}^0}\right)^{1/n} + nkT\left(\frac{-\Delta E}{nkT}\right) \tag{5}$$

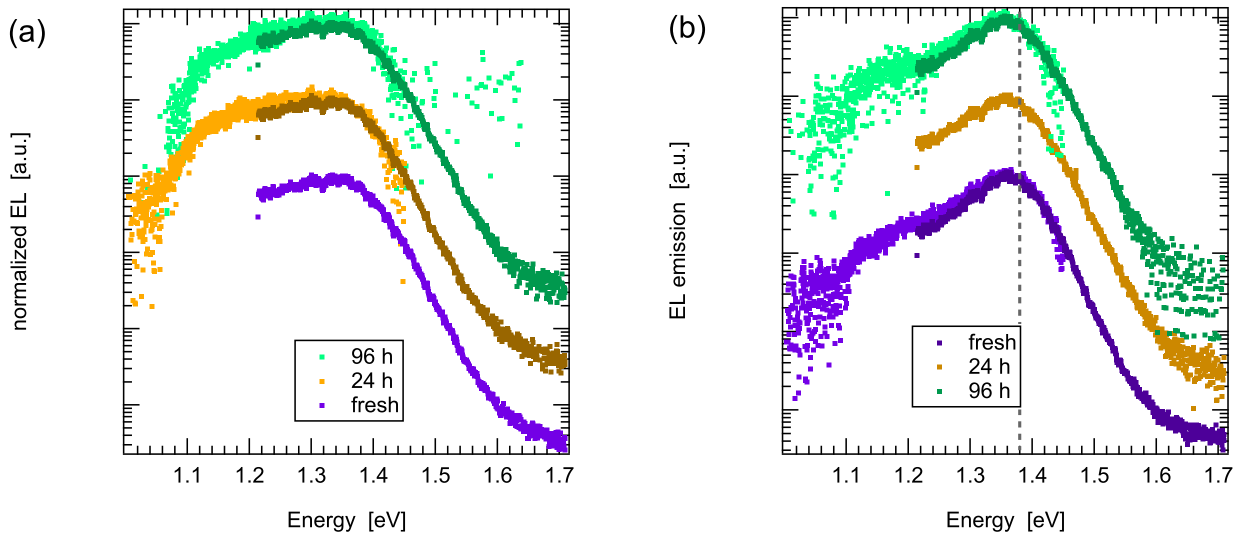

Supplementary Figure 4. Normalized electroluminescence spectra. (a) 100 nm and (b) 200 nm thick device at room temperature under constant injection current density of 96 mA cm^{-2}. The spectra were measured by liquid-Nitrogen cooled Indium-Gallium-Arsenide- and Silicon-cameras and were subsequently normalized to match at their maximum around 1.35 eV.

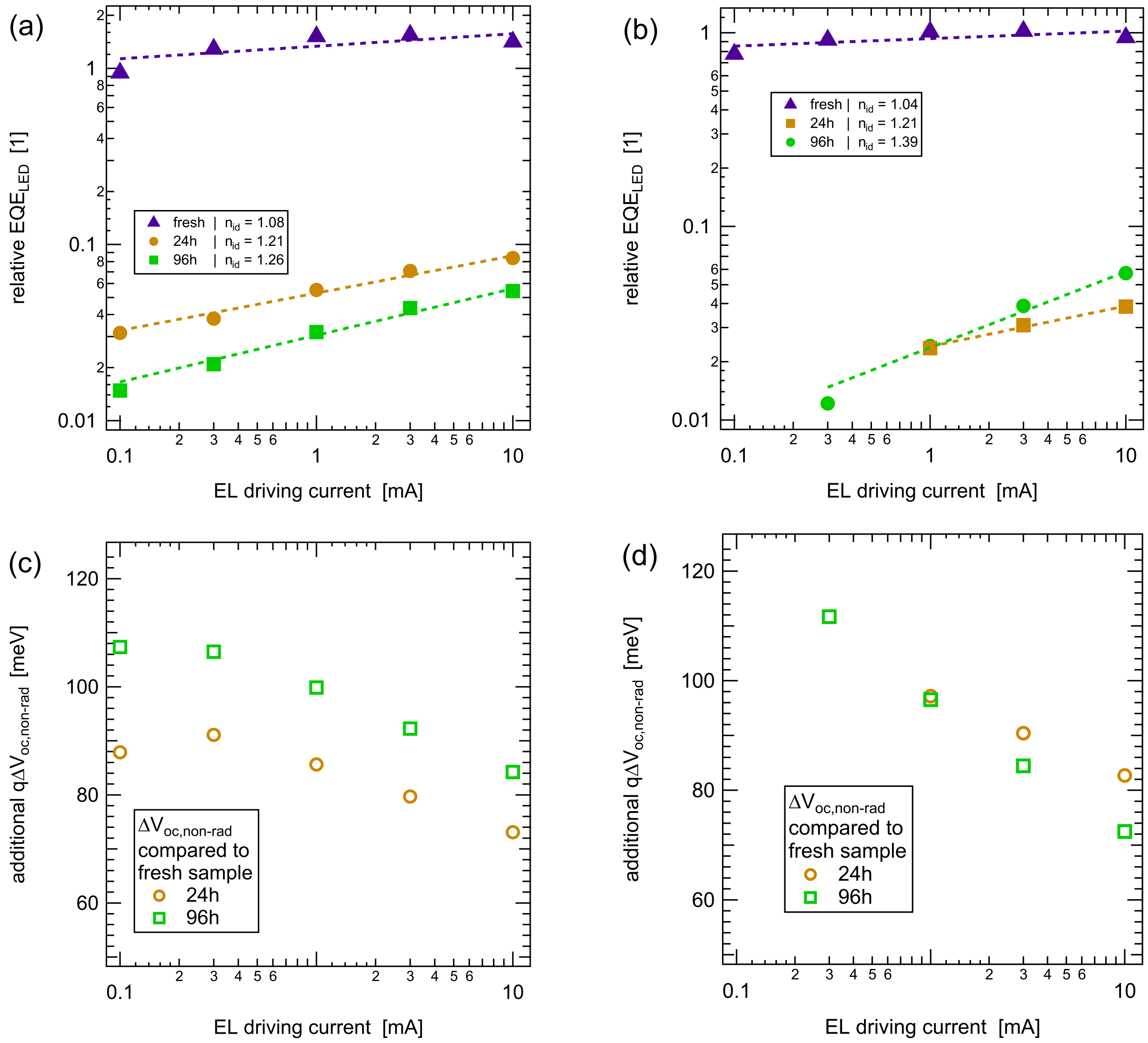

Supplementary Figure 5. Integrated electroluminescence spectra. (a) 100 nm and (b) 200 nm thick devices at room temperature and increasing injection currents. The slope of the integrated values (dashed line) corresponds to the electroluminescence ideality factor, which increases during aging. At the same time, the overall electroluminescence intensity is reduced upon aging, suggesting increased non-radiative recombination. The lower integral value corresponds to an additional non-radiative voltage loss for the (c) 100 nm and (d) 200 nm thick device. However, as the devices have to be kept under inert atmosphere for the 96 h aging procedure and are placed in a custom-build sample chamber for electroluminescence measurements, the absolute emission quantum yield could not be determined with reasonable certainty.

Effective band gap. Recently, it was found from electroluminescence measurements that while overall recombination in PM6:Y6 occurs non-radiatively from CT states, the radiative recombination is dominated by S_1 states in Y6, which are repopulated via an energy barrier of $\Delta E_{\mathrm{S_1\text{-}CS}} \approx 117$ meV.[3] As a result, the EL quantum yield is temperature dependent with an exponential dependency on $\Delta E_{\mathrm{S_1\text{-}CS}}$ and kT.
The repopulation of S_1 states causes a reduced quasi-Fermi-level splitting of the charge-separated states at high temperatures. According to Eqn. (5), this energy loss can be attributed to the energy barrier $\Delta E_{\mathrm{S_1\text{-}CS}}$ and affects the measured V_{oc} of the device:

$$\mathrm{QY_{EL}} = \mathrm{QY_{EL}^0} \exp\left(-\frac{\Delta E_{\mathrm{S_1\text{-}CS}}}{kT}\right) \tag{6}$$

$$eV_{\mathrm{oc}} \approx n_{\mathrm{id}} kT \ln\left(J_{\mathrm{sc}}\right) - kT \ln\left(\frac{J_0^{\mathrm{rad}}}{\mathrm{QY_{EL}^0}}\right) - \Delta E_{\mathrm{S_1\text{-}CS}} \tag{7}$$

The extrapolated limit of V_{oc} to 0 K, $E_{\mathrm{g,eff}}$, is therefore always lower than the (ideal) radiative quasi-Fermi-level splitting (QFLS) suggests, and $\Delta E_{\mathrm{S_1\text{-}CS}}$ can be expressed as a temperature-independent part of the non-radiative V_{oc} losses ($kT \ln\left(\mathrm{QY_{EL}}\right)$). While the QFLS limit was found at 1.1 eV, the corrected effective bandgap (defined by the limit of the radiative recombination current J_0^{rad} and QY_{EL}^0) is located at $E_{\mathrm{g,eff}} + \Delta E_{\mathrm{S_1\text{-}CS}} \approx 1.22$ eV according to Eqn. (7). However, the origin of the remaining difference of 160 meV between the effective band gap and the optical band gap still remains unclear.

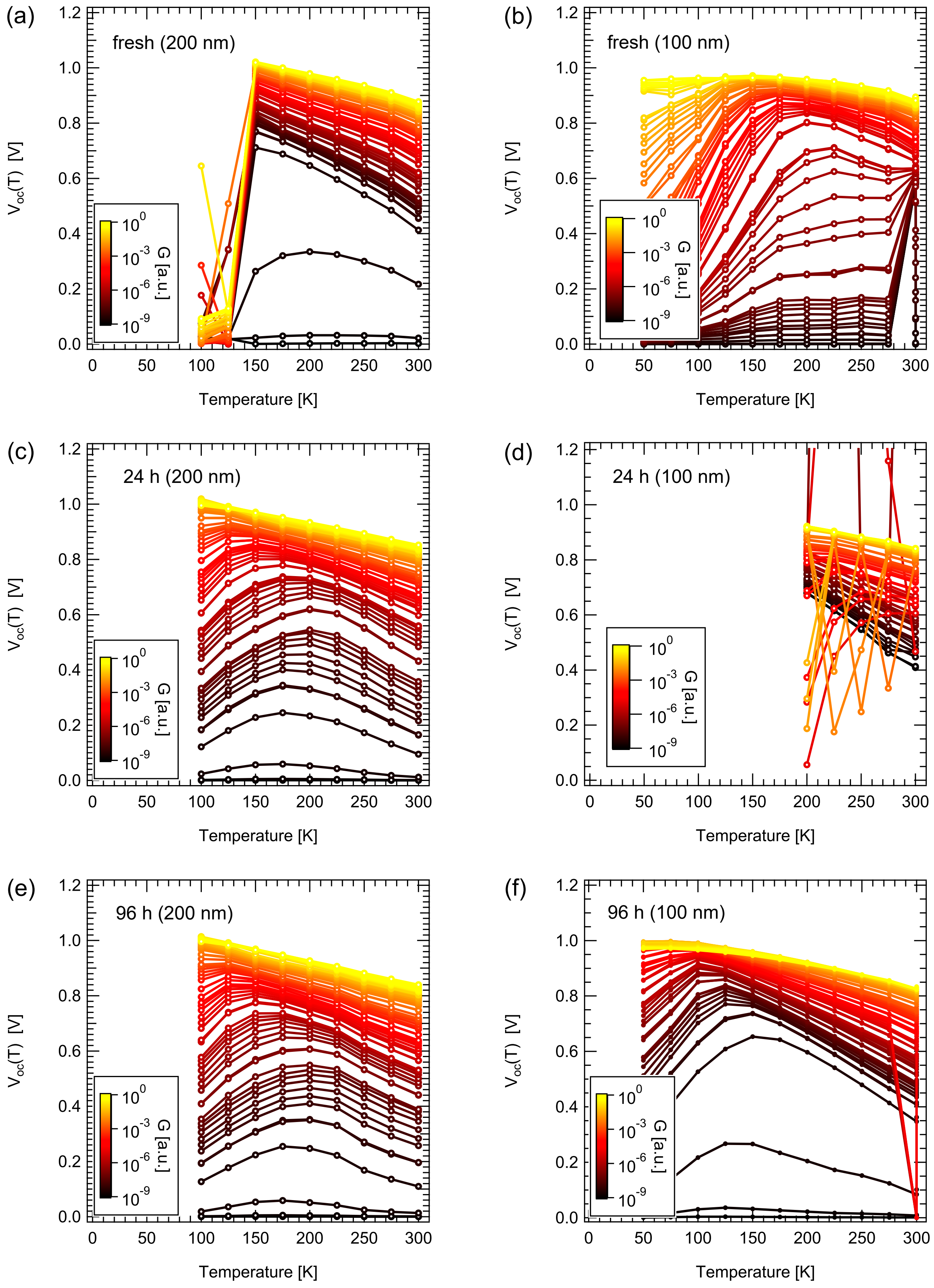

Supplementary Figure 6. Open circuit voltages V_{oc} for temperature T and for 9 orders of magnitude of generation rate G of 200 nm thick device (a) fresh, (c) aged for 24 h, (e) aged for 96 h, and 100 nm think device (b) fresh, (d) aged for 24 h, (f) aged for 96 h. The measured values are shunt-limited at very low generation rates, and contact issues occur in the custom-built cryostat at low temperatures.

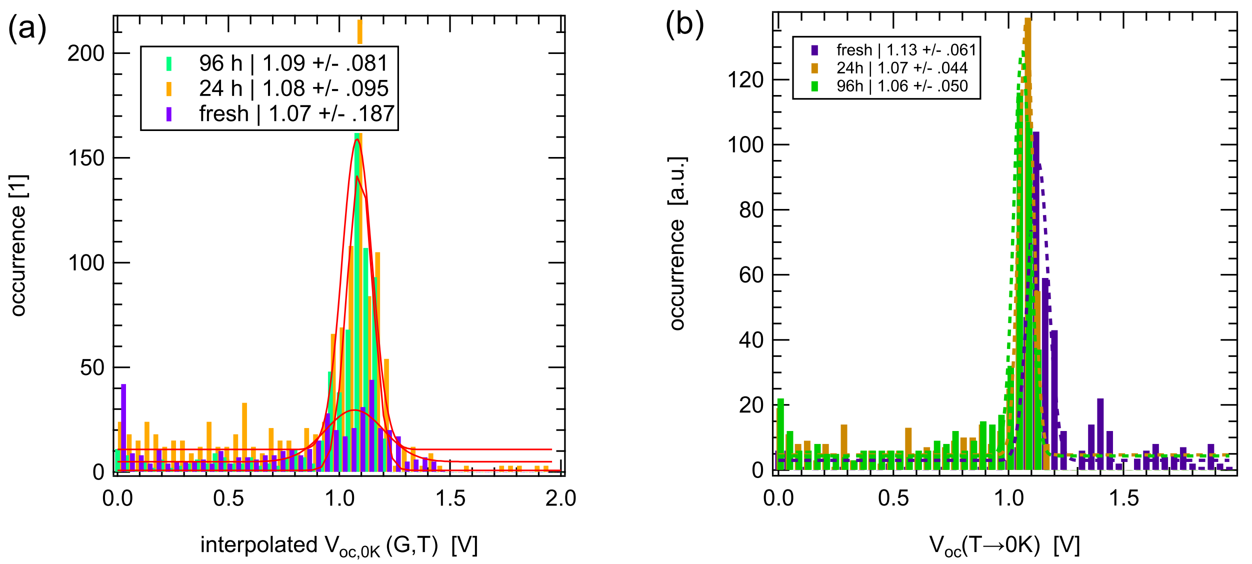

Supplementary Figure 7. Histograms of open-circuit voltages (Fig. 6), locally linear extrapolated to $T = 0$ K. (a) 100 nm and (b) 200 nm thick devices at different aging steps. The histograms were fitted with a normal distribution to determine the low temperature limit of the open-circuit voltage. Values for the limit and uncertainties from the distribution width are given in the annotations; no significant change is caused by the aging process.

Supplementary Note IV. Conductivity

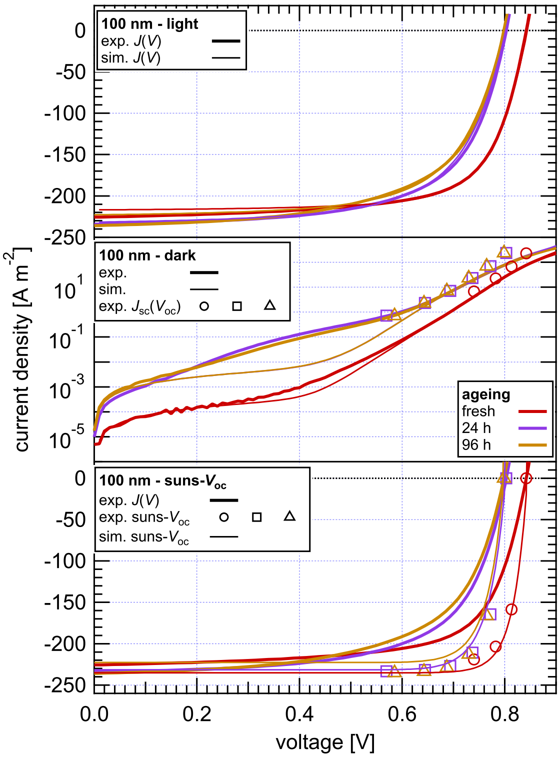

Supplementary Figure 8. Current–voltage characteristics of fresh and aged solar cells with an active-layer thickness of 100 nm. (Top) Experimental and simulated JV curves under 1-sun equivalent illumination. (Center) Experimental JV curves in the dark and $J_{\mathrm{sc}}(V_{\mathrm{oc}})$ data as well as simulated JV curves in the dark. The experimental data show a parasitic feature at the transition from the shunt resistance-dominated part to the exponential part ranging from 0.1 to 0.6 V. Although its origin is unknown, we assign it to sample-to-sample variation, as other pixels on the same substrate did not show such behavior. Therefore, data in the voltage region mentioned above were excluded from the simulation. (Bottom) The same experimental JV curves as in the top graph together with experimental and simulated suns-V_{oc} data, showing a significant voltage difference ΔV between JV and suns-V_{oc} data at a given current density. The conductivity σ extracted from ΔV is 9.4, 8.1 and $6.5 \cdot 10^{-5}$ $\mathrm{S\,m^{-1}}$ for aging durations of 0, 24, and 96 h, respectively.

Supplementary Note V. Capacitance–voltage measurements

We evaluated experimental CV curves to achieve the effective charge carrier density n as well as an estimate for the intrinsic charge carrier density n_i under open-circuit conditions.
Recently, several commonly used approaches for estimating $n\left(V_\mathrm{sat}\right)$ and C_μ were compared.[4] We chose to determine $n\left(V_\mathrm{sat}\right)$ and C_μ by

$$C_\mu\left(V\right) = C\left(V\right) - C_\mathrm{geo} \tag{8}$$

$$n\left(V_\mathrm{sat}\right) = \frac{1}{eL}\left(V_\mathrm{oc} - V_\mathrm{sat}\right) \tag{9}$$

with C_geo being the geometrical capacitance. V_sat is a voltage in the reverse-bias regime, where non-geminate recombination is heavily suppressed. n is obtained from the chemical capacitance $C_\mu\left(V\right)$ using the equation

$$n\left(V_\mathrm{oc}\right) = n\left(V_\mathrm{sat}\right) + \frac{1}{eL}\int_{V_\mathrm{sat}}^{V_\mathrm{oc}} C_\mu\left(V\right)\mathrm{d}V \quad . \tag{10}$$

With n being known as a function of V_oc, the ideality factor n_n of charge carrier density is calculated. Since $n\left(V_\mathrm{oc}\right)$ follows[5]

$$n = n_0 \exp\left(\frac{eV_\mathrm{oc}}{n_n kT}\right) \quad , \tag{11}$$

we can determine n_n by locally deriving the natural logarithm of n.
With $n\left(V_\mathrm{oc}\right)$ being known we achieve an estimate for n_i of the active layer blend[6]

$$n_\mathrm{i} = n\left(V_\mathrm{oc}\right)\exp\left(-\frac{eV_\mathrm{oc}}{2kT}\right) \quad . \tag{12}$$

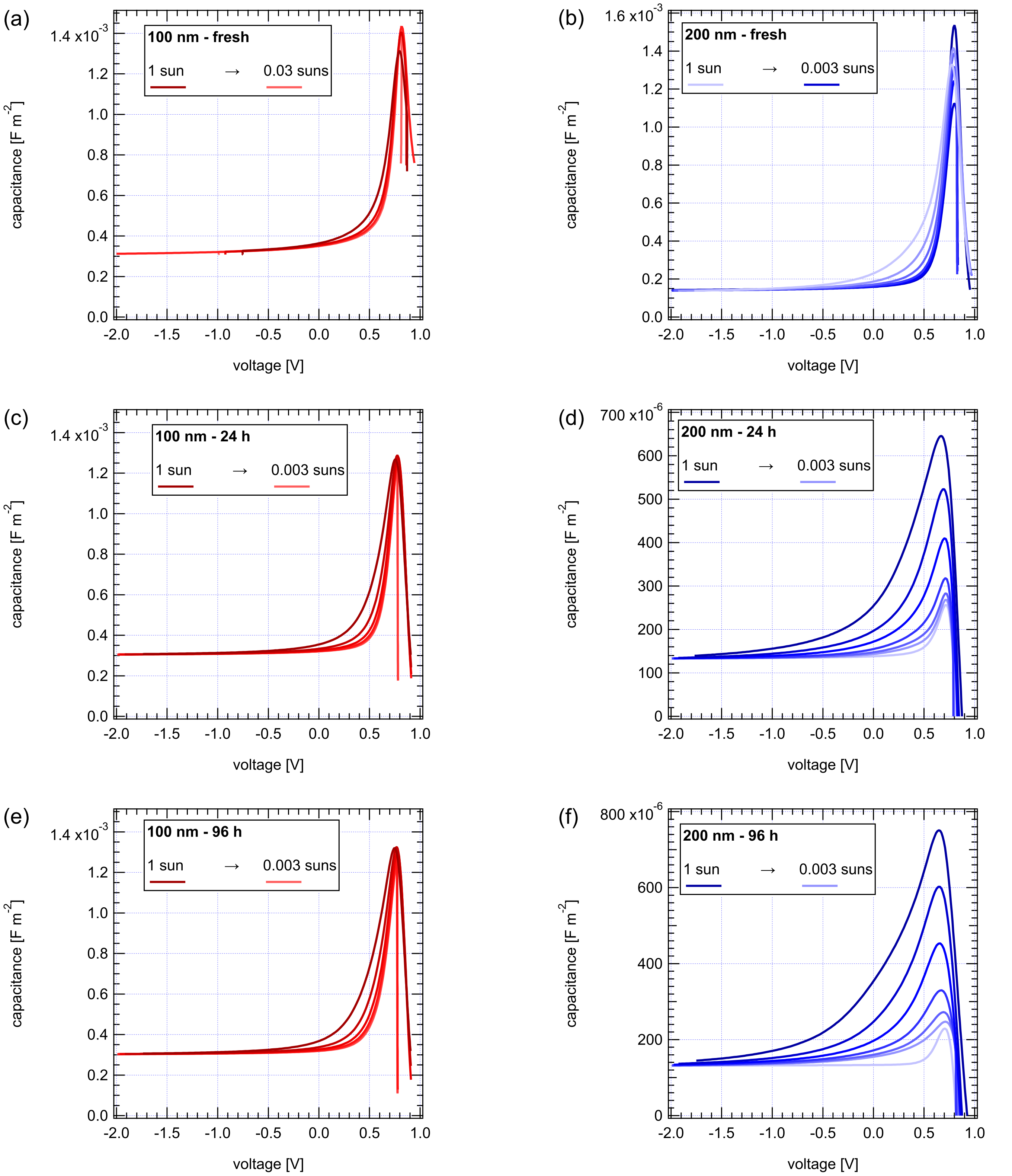

Supplementary Figure 9. Capacitance–Voltage (CV) measurements of 100 nm thick device (a) fresh, (c) aged for 24 h, (e) aged for 96 h, and 200 nm think device (b) fresh, (d) aged for 24 h, (f) aged for 96 h.

Supplementary Note VI. Intensity-modulated spectroscopy

The Nyquist representations of IMVS data (Fig. 10) generally show one complete semicircle in the second quadrant and one incomplete semicircle in the third quadrant, both exhibiting decreasing radii with decreasing illumination intensity. The incomplete semicircles, occurring at small frequencies of 1 to 10 Hz, are attributed to parasitic effects, most probably originating from the setup and therefore ignored in the evaluation.

In the Bode representation (Fig. 10) of the IMVS real part, a plateau occurs at intermediate angular frequencies between 100 and 1000 Hz. Because the imaginary part at such small frequencies is almost zero, the real part can be used as an approximation for the voltage response V_{AC} to the modulated intensity amplitude G_{AC}, which we chose to be 10 % of the bias illumination amplitude G. The diode ideality factor n_{id} can be calculated from the IMVS real part according to Eqn. (1) in the main text.

The effective small-perturbation charge-carrier lifetime $\tau_{\Delta n}$ is extracted from the angular frequency, at which the imaginary part of the IMVS occurs. With the recombination order λ (see below) this can be transformed into the effective charge-carrier lifetime $\tau_n = (\lambda + 1)\,\tau_{\Delta n}$ shown in Fig. 11. Both $\tau_{\Delta n}$ and τ_n follow[5]

$$\tau_n = \tau_{n,0} \exp\left(-\frac{eV_{\mathrm{oc}}}{n_\tau kT}\right) \quad . \tag{13}$$

From here, the lifetime ideality factor n_τ is calculated by locally deriving the natural logarithm of the above equation. The results are shown in Fig. 11. For moderate and small illumination intensities below 0.1 suns, n_τ is generally dropping with aging, ranging from 2.3 for the fresh device to 1.8 for the 96 h aged one. At higher illumination intensities approaching 1 sun, n_τ drops below 1.5 for all aging steps studied here, which we again attribute to surface recombination. At 0.1 suns and below n_τ approaches and saturates around a value of 2.

The recombination ideality factor. The recombination ideality factor n_{R}, which was shown to be equal to n_{id},[5] is calculated as the harmonic mean of n_n and n_τ. Then, n_{R} is compared to n_{id} calculated from IMVS (Fig. 11). Although both ideality factors are not exactly equal, they display a comparable trend: n_{R} decreases with increasing illumination intensity, starting at approximately 1.4 for 0.003 suns and approaching unity at 0.1 suns for all aging steps. At illumination intensities above 0.3 suns, it drops below 1, which is again assigned to surface recombination becoming dominant.

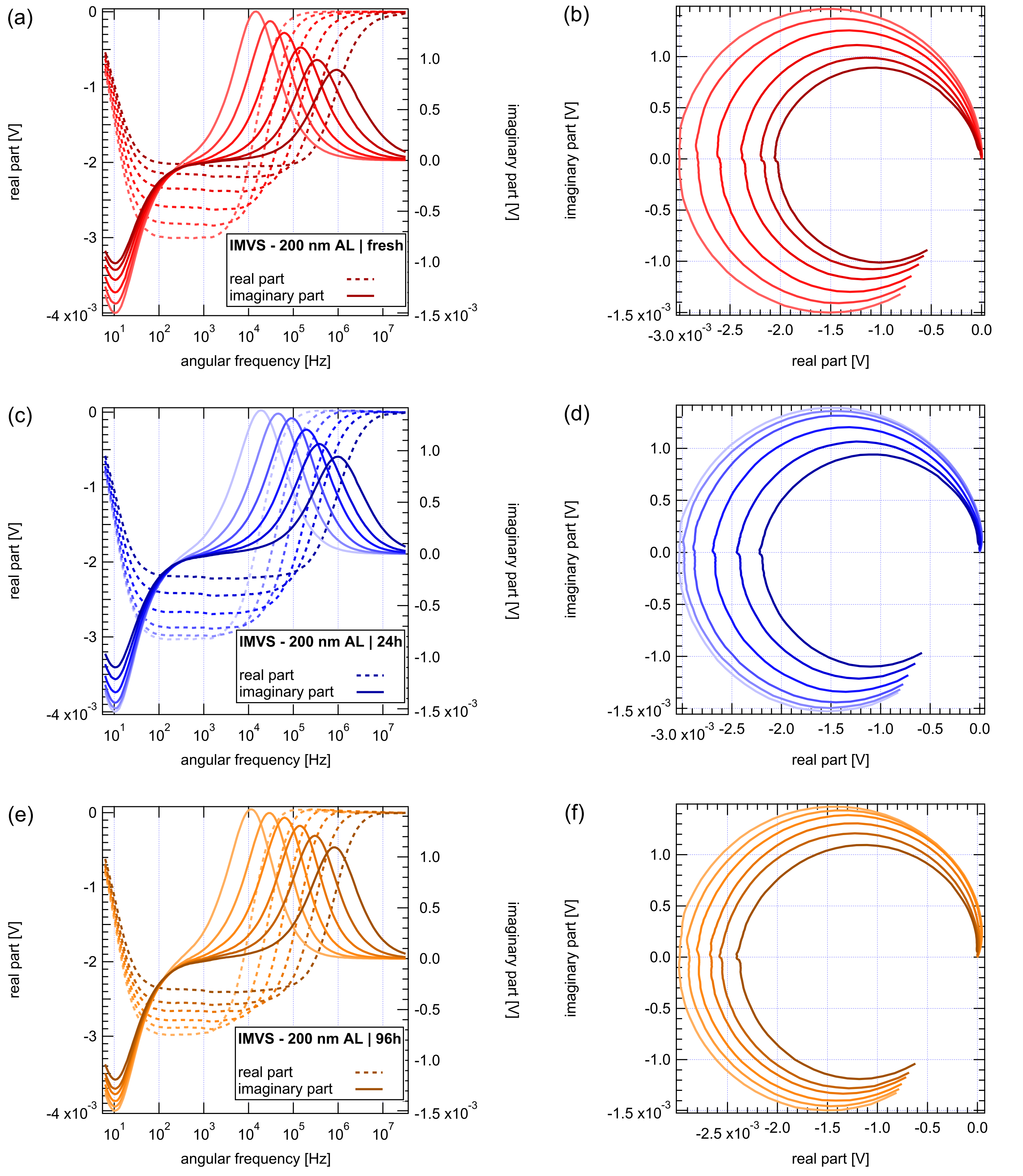

Supplementary Figure 10. IMVS raw data. Bode representation of (a) fresh 200 nm thick device, (c) aged for 24 h, (e) aged for 96 h. Nyquist representation of (a) fresh 200 nm thick device, (c) aged for 24 h, (e) aged for 96 h.

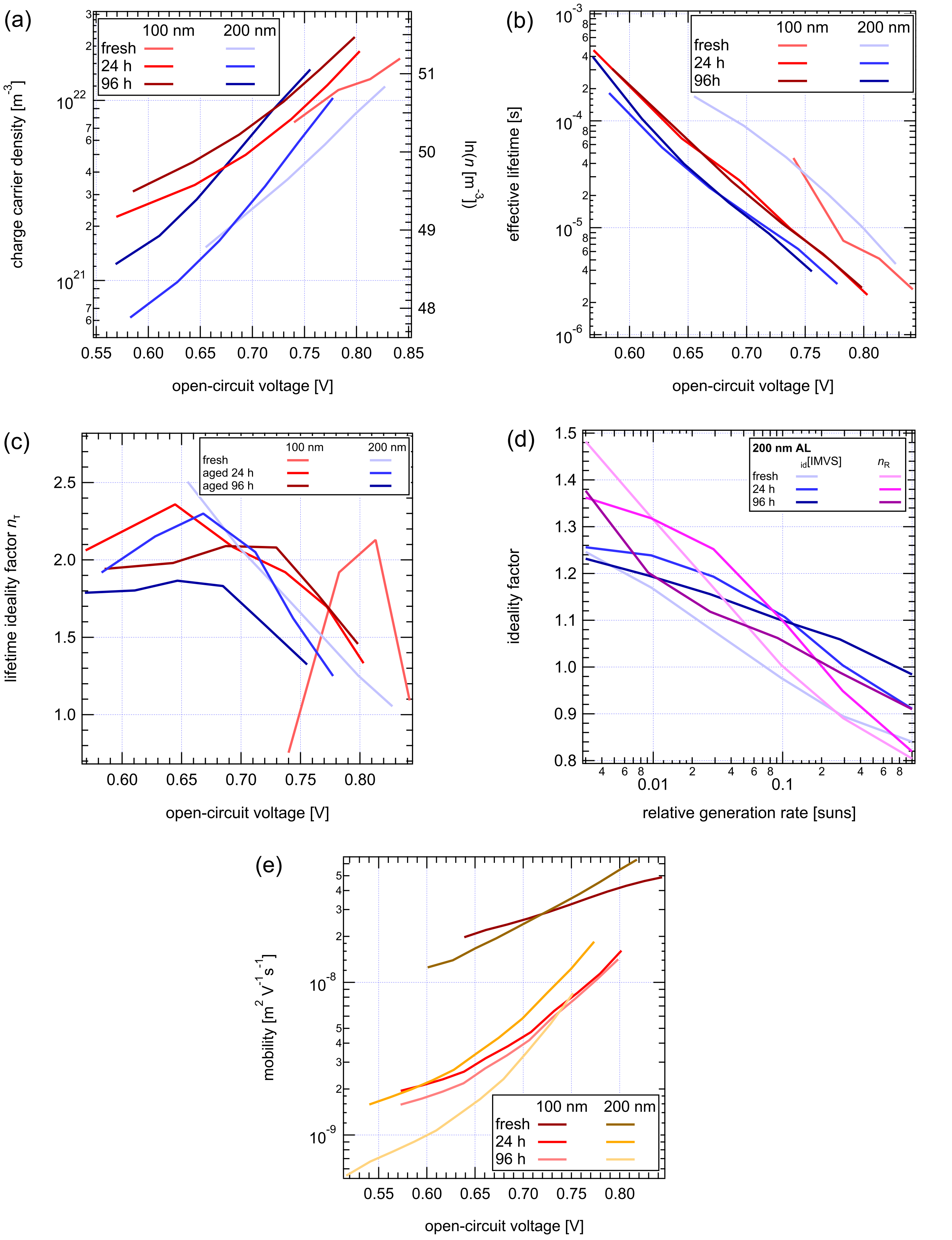

Supplementary Figure 11. Parameters derived from electro-optical measurements. (a) Effective charge-carrier density n. (b) effective charge carrier lifetime τ_n for subsequent aging steps. (c) n_τ extracted from the measurements of τ_n. (d) device ideality factor $n_{\rm id}$ and recombination ideality factor $n_{\rm R}$ for the thick device. (e) Simulated charge carrier mobility.

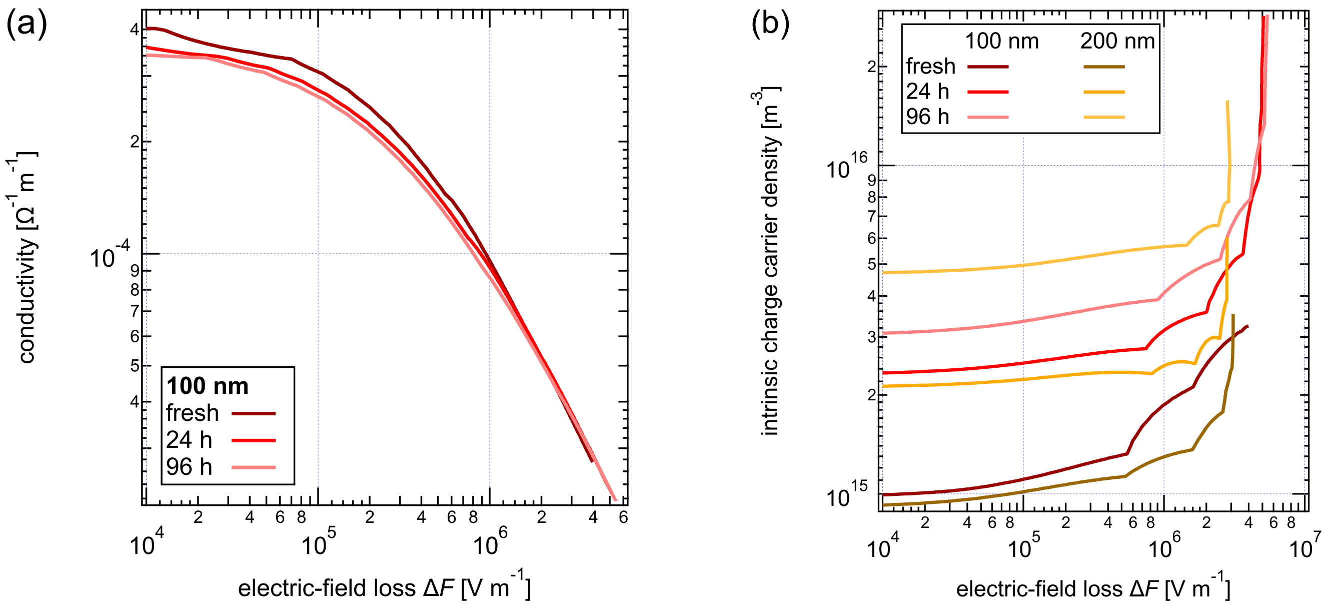

Supplementary Figure 12. Field-dependent electric conductivity and intrinsic charge carrier density. (a) σ of the device with 100 nm thick active layer, (b) intrinsic n_i. The electric-field screening ΔF is determined from JV measurements under 1 sun-equivalent monochromatic illumination and suns–V_oc measurements for each current density.

Supplementary Note VII. Grazing Incidence Wide Angle X-Ray Scattering (GIWAXS)

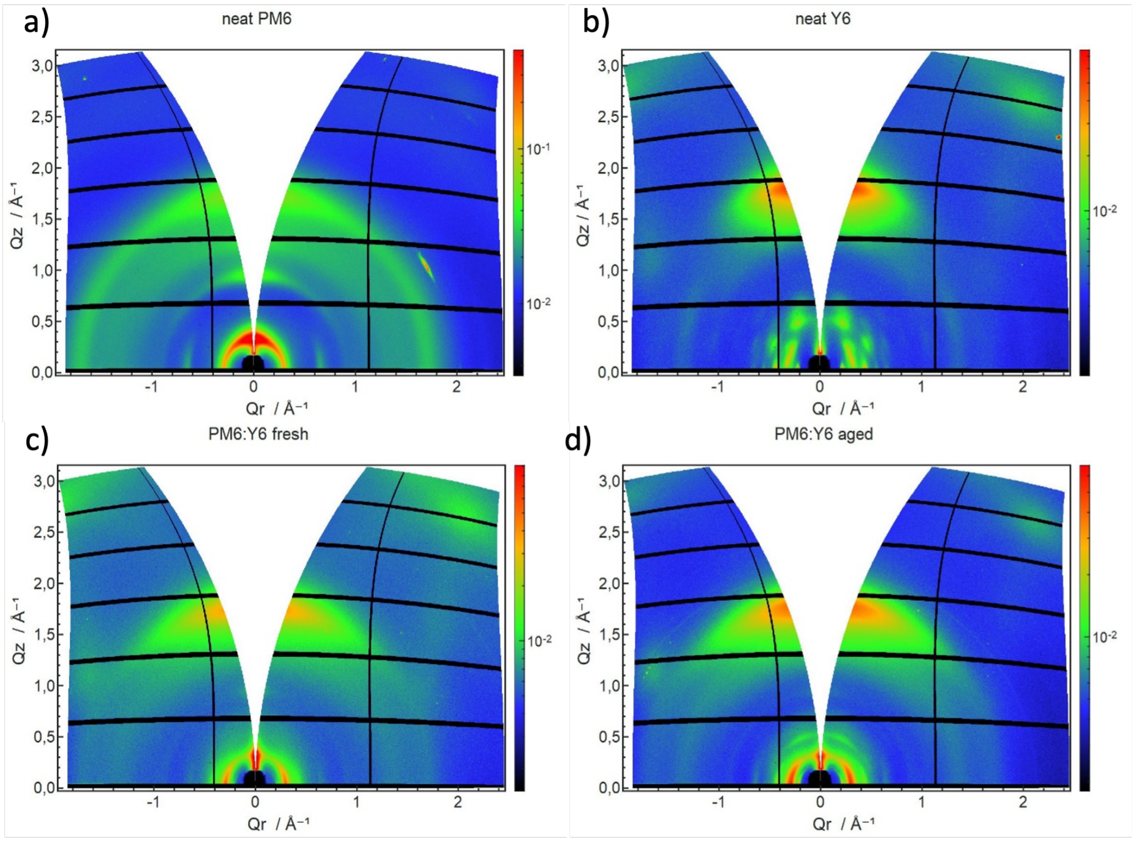

Supplementary Figure 13. q-converted 2D GIWAXS data for samples on silicon substrates. a) PM6, b) Y6, c) PM6:Y6 fresh and d) PM6:Y6 aged for 96 h spincoated on silicon substrates. Vertical information along q_z (region around the vertical gap) includes information about orientation perpendicular to substrate, whereas horizontal information along q_r holds information on orientation parallel to the substrate.

The samples of neat PM6 and neat Y6 thin films spincoated on silicon substrates show each high ordering in the 2D GIWAXS data (Fig. 13a and b). Upon blending both materials, the GIWAXS data shows a reduction in the long range ordering for both materials (Fig. 13c). However, during the implemented ageing process both material components recover some of their neat material order signatures (Fig. 13d). Particularly for the Y6 the peaks observed in the lower q-range indicate that several molecules must arrange in lateral and vertical direction.

The q-converted 2D GIWAXS data for a) PM6, b) Y6, c) PM6:Y6 fresh and d) PM6:Y6 aged for 96 h on ITO/ZnO are shown in Fig. 14. In contrast to the PM6:Y6 blends prepared on silicon substrates the ones prepared on ITO coated with ZnO show already weaker ordering for the neat materials (Fig. 14a and b) and a much weaker dependence on aging (Fig. 14c to d). The π–π-peak of the neat PM6 is a Lorentzian with $q_{z,f}$ = (16.70 ± 0.02) nm^{-1} with a real space distance of $d_{v,f}$ = (3.762 ± 0.006) Åand the π–π-peak of the neat Y6 is a Lorentzian with $q_{z,f}$ = (17.36 ± 0.04) nm^{-1} with a real space distance of $d_{v,f}$ = (3.619 ± 0.009) Å. The π–π-peak of the blend can be fit with a variety of combinations of these two peak contributions depending of the allowed degrees of freedom (peak height, peak width, peak position). In all cases the PM6 contribution dominates strongly and shows a clear change from a more well defined Lorentz to a broader peak after 96 h of artificial aging.

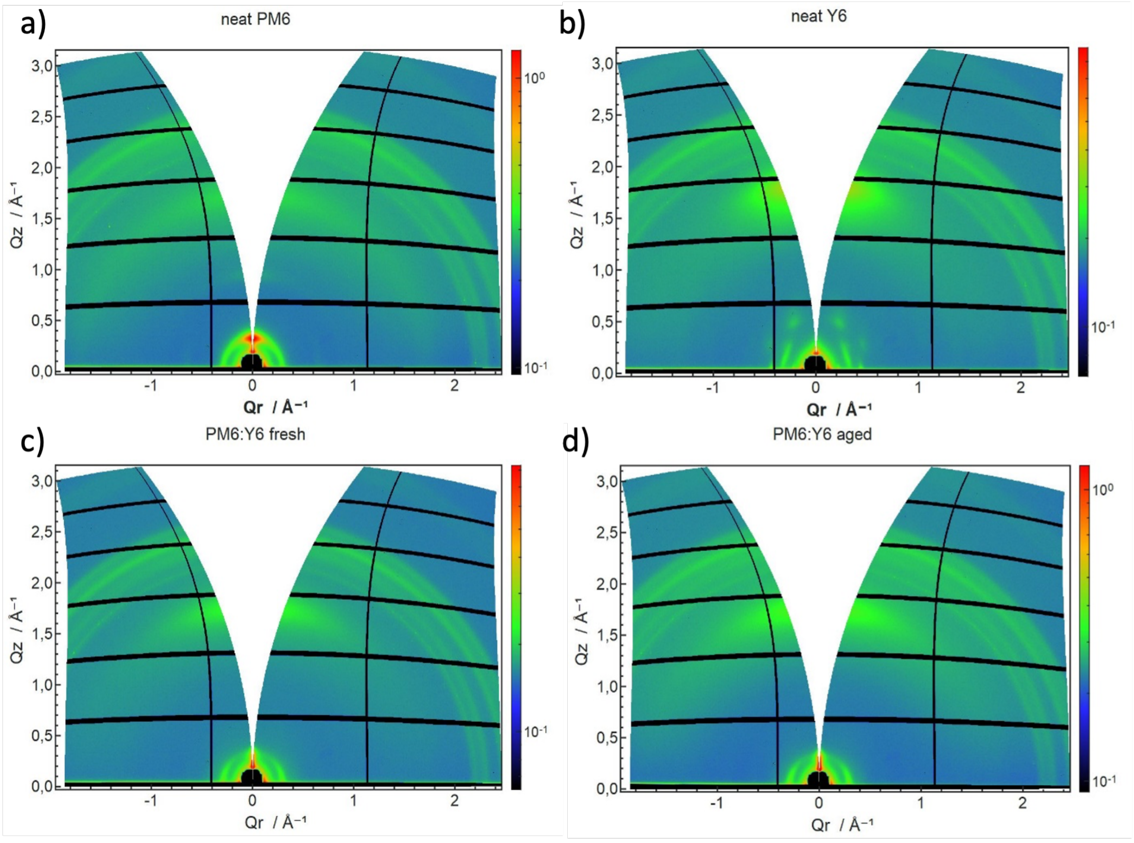

Supplementary Figure 14. ***q*-converted 2D GIWAXS data for samples on ZnO-coated ITO substrates.** a) PM6, b) Y6, c) PM6:Y6 fresh and d) PM6:Y6 aged for 96 h spincoated on ITO/ZnO coated glass substrates. 1D intensity data (Fig. 1c) was extracted with two cuts, containing horizontal and vertical information. Vertical information (15°azimuthal integration around the vertical gap) includes information about orientation perpendicular to substrate, whereas horizontal information (15°azimuthal integration parallel to x-axis) holds information on orientation parallel to the substrate.

Supplementary Note VIII. Ultraviolet photoemission spectroscopy (UPS)

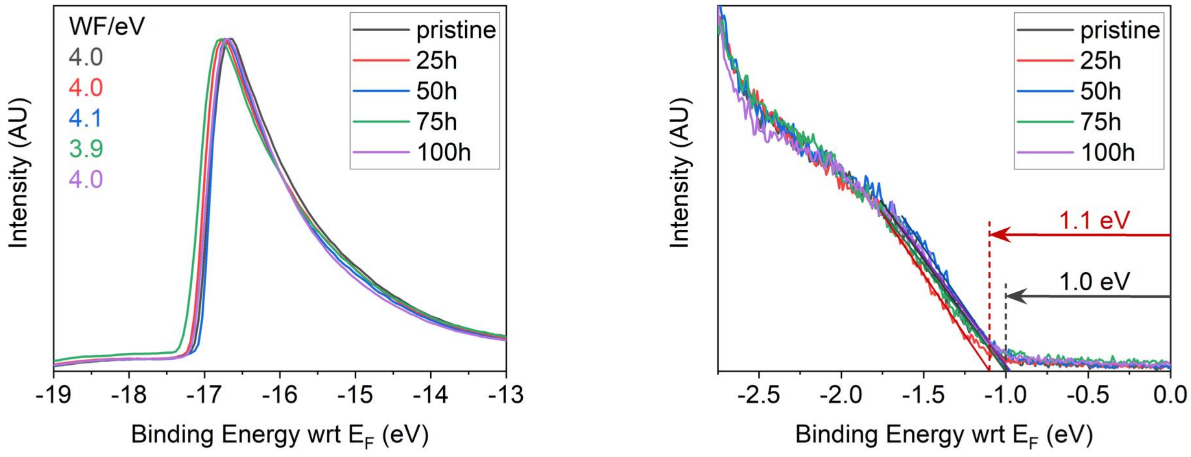

Supplementary Figure 15. UPS traces on surfaces of fresh (pristine) and aged samples.

Supplementary Note IX. Details of the device simulation

The model. The details of the device model were described elsewhere, in short the model solves the drift–diffusion equations, Poisson's equation, and the Shockley-Read-Hall (SRH) trapping and escape equations. It is important to note that the SRH capture escape equations are solved explicitly. This means that the device is discretized in position space using a finite difference mesh, the transport and drift diffusion equations are solved on this mesh between the contacts. Under each mesh point in position space, energy space is also discretized, meaning the LUMO and HOMO are split up into 5-10 distinct energy levels. For each of these energy ranges a SRH capture escape equation is solved. The capture escape rates are calculated before the simulation begins by integrating the SRH functions over the exponential distribution of trap states for each energy range, then tabulating them as a function of trap Fermi level and free carrier Fermi level. This approach allows dynamic carrier events to be simulated, which is distinctly different to simply solving the analytical SRH equation already integrated over energy space at each mesh point.

Extension of the model to frequency domain. Previously the general approach to solving frequency domain problems was to solve the DC problem then cast the same set of equations into a small signal form,[7] solve these and add the two results. This approach although efficient has two drawbacks. The first is casting the Jacobian into a small signal form is often nontrivial to do, and one effectively ends up maintaining two Jacobians, one DC/time domain form and one small signal one. The second issue with this approach is that it is not clear when/where the linear approximation is valid, this is an especially relevant concern when one is dealing with a non-linear set of traps. Considering the progress in multi-core computers since the work by Laux,[7] we decided for this work to model the frequency domain by solving the set of equations in the time domain for each frequency of interest. Therefore to simulate impedance spectroscopy voltage perturbations were applied to the time domain model and the resulting current examined. Each simulation is run on its own thread in parallel.

Extracting phase information from AC signals. Extracting real and imaginary phase information from simulated AC signals is not straightforward, because the signal is comprised of a finite number of points. To extract the phase information exactly, one would need a very fine time step, which would be costly to simulate. Instead in this work, we used as few time domain points as necessary to simulate the signal accurately, then extract the real and imaginary parts by fitting a perfect sine wave to the simulated AC output/input signals.

The fit is performed by first taking the discrete Fourier transform of the signals to get an estimate of the phase and magnitude, the result is not accurate due to the non-infinite nature of the signal. We then use the phase information to guess where to place the sine functions on top of the simulated data to get a good fit. Newton's method is then used to optimize the magnitude, phase, and DC offset in turn.

Fitting to experimental data. Tables I and II show the simulation parameters for the 100 nm and 200 nm thick devices. Values below the horizontal line were constants, those above the line were allowed to vary. The results of the fits can be seen in Figs. 16–18 for the 100 nm device and in Figs. 19–21 for the 200 nm device. The quality of the fit was prioritized in the following order: dark JV curve, light JV curve, CV, Suns-V_{oc}, impedance spectroscopy data. The downhill simplex algorithm was used for the fitting, each simulation (including each frequency domain point) was run of an individual thread.

Supplementary Table I. Simulation parameters for an active-layer thickness of 200 nm.

Parameter	0 h	24 h	96 h	Units
Electron trap density	1.1×10^{25}	2.6×10^{25}	3.3×10^{25}	m^{-3} eV^{-1}
Hole trap density	6.7×10^{23}	1.3×10^{25}	1.6×10^{25}	m^{-3} eV^{-1}
Free hole to Trapped electron	9.6×10^{-23}	3.9×10^{-23}	2.6×10^{-23}	m^{-2}
Free electron to Trapped hole	1.3×10^{-23}	3.9×10^{-23}	2.6×10^{-23}	m^{-2}
Free hole to Trapped hole	2.8×10^{-21}	1.6×10^{-21}	1.9×10^{-21}	m^{-2}
Free electron to Trapped electron	1.3×10^{-23}	1.6×10^{-21}	1.9×10^{-21}	m^{-2}
Series resistance	0.270	0.161	5.956	*Ohms*
Contact charge density	2.4×10^{23}	1.2×10^{23}	1.2×10^{23}	m^{-3}
Charge carrier mobility	6.8×10^{-08}	1.3×10^{-07}	1.5×10^{-07}	$m^2V^{-1}s^{-1}$
Electron tail slope	0.065	0.065	0.065	eV
Hole tail slope	0.075	0.075	0.075	eV
Photon efficiency	0.677	0.704	0.674	$0 - 1$
Shunt resistance	1.4×10^{03}	80.000	80.000	$Ohmsm^2$
E_g	1.250	1.250	1.250	eV

Supplementary Table II. Simulation parameters for an active-layer thickness of 100 nm.

Electron trap density	1.9×10^{24}	2.3×10^{25}	6.8×10^{25}	m^{-3} eV^{-1}
Hole trap density	2.5×10^{24}	1.2×10^{25}	2.7×10^{25}	m^{-3} eV^{-1}
Free hole to Trapped electron	3.0×10^{-25}	6.0×10^{-23}	4.1×10^{-23}	m^{-2}
Free electron to Trapped hole	3.2×10^{-23}	6.0×10^{-23}	4.1×10^{-23}	m^{-2}
Free hole to Trapped hole	3.8×10^{-18}	1.3×10^{-21}	9.4×10^{-21}	m^{-2}
Free electron to Trapped electron	1.2×10^{-22}	1.3×10^{-21}	9.4×10^{-21}	m^{-2}
Series resistance	0.015	0.357	4.276	*Ohms*
Contact charge density	8.1×10^{22}	8.1×10^{22}	8.1×10^{22}	m^{-3}
Charge carrier mobility	1.1×10^{-07}	2.1×10^{-07}	2.8×10^{-07}	$m^2V^{-1}s^{-1}$
Electron tail slope	0.065	0.065	0.065	eV
Hole tail slope	0.075	0.075	0.075	eV
Photon efficiency	0.645	0.648	0.692	$0 - 1$
Shunt resistance	500.000	130.000	300.000	$Ohmsm^2$
E_g	1.250	1.250	1.250	eV

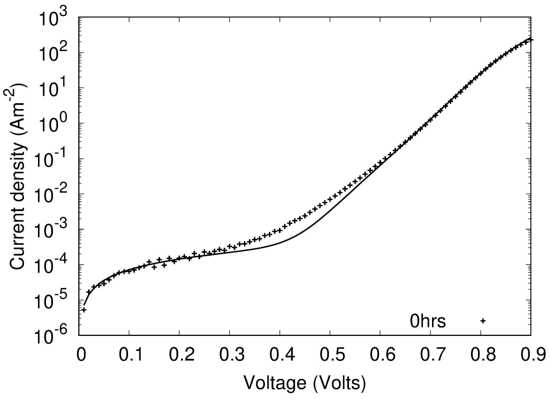

(a) Dark JV

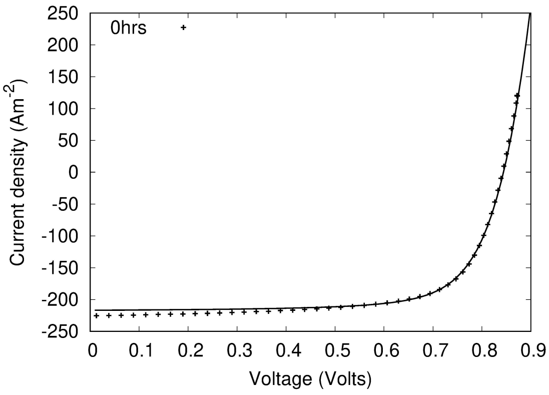

(b) Light JV

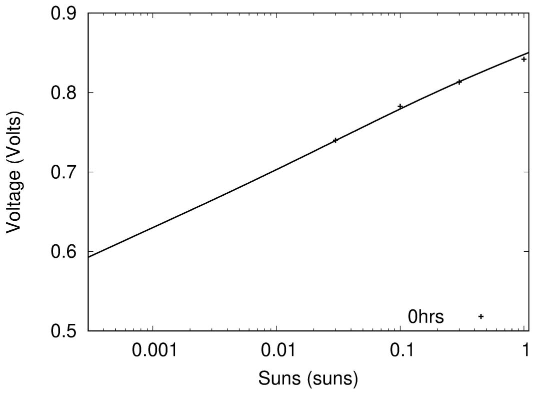

(c) Suns–V_{oc}

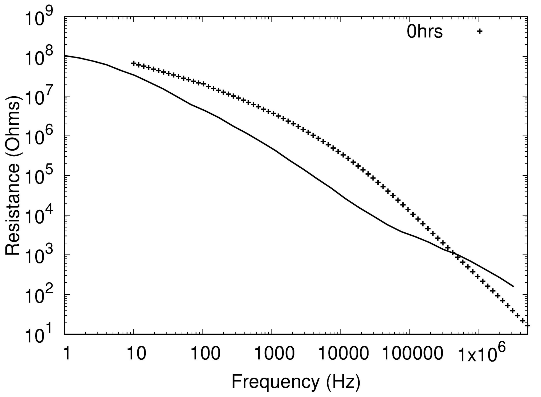

(d) Real part of IS spectra.

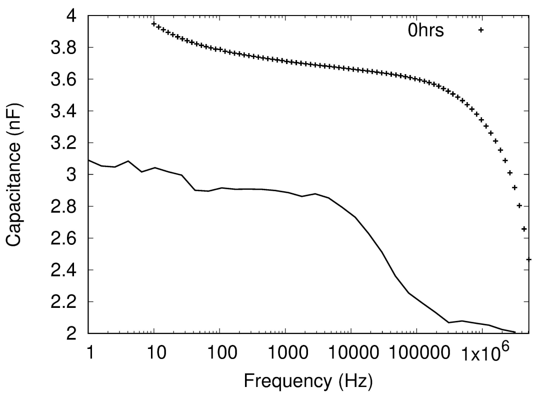

(e) Imaginary part of IS spectra.

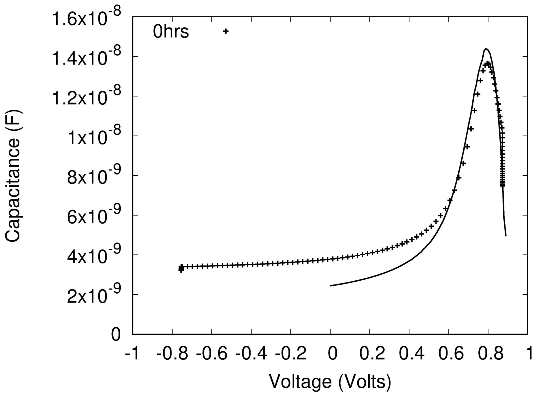

(f) Capacitance–Voltage

Supplementary Figure 16. Fits of the model to the 100 nm thick fresh device for (a) dark JV, (b) light JV, (c) suns–V_{oc}, (d) real part of IS spectra, (e) imaginary part of IS spectra, and (f) capacitance–voltage experimental data.

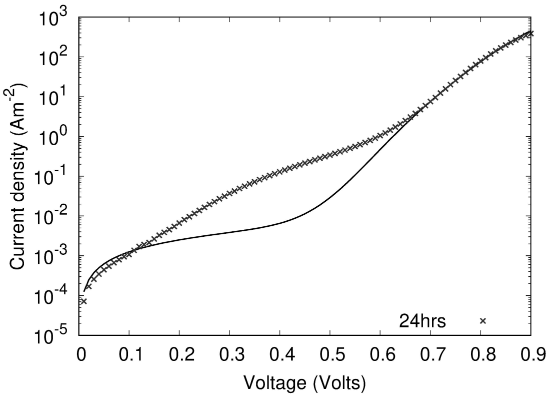

(a) Dark JV

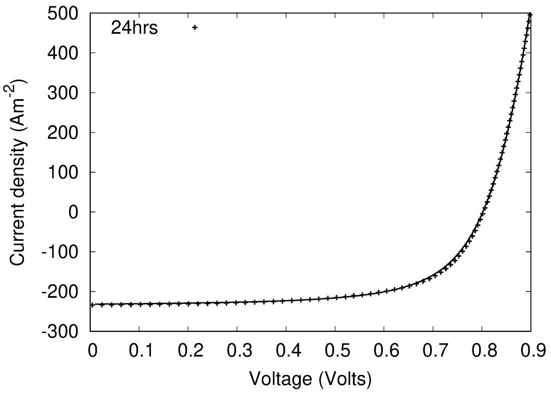

(b) Light JV

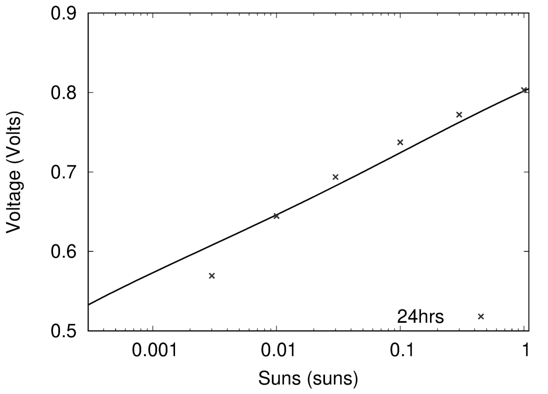

(c) Suns–$V_{\rm oc}$

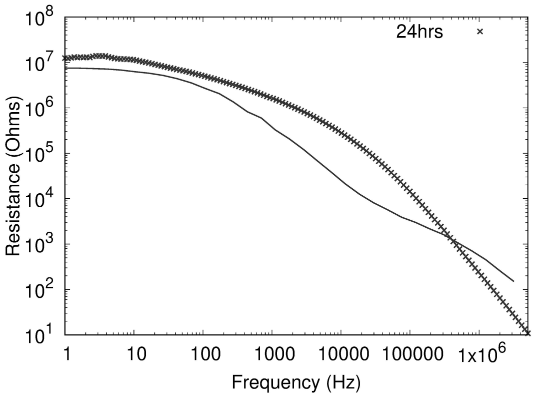

(d) Real part of IS

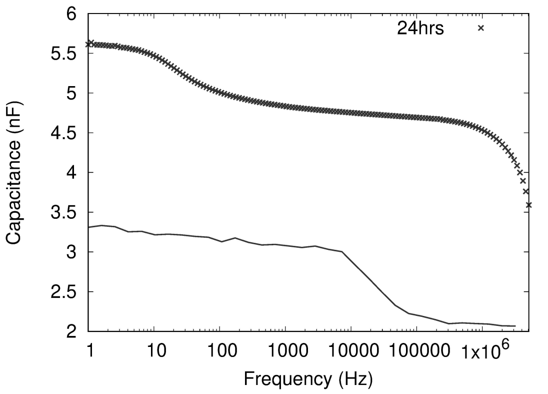

(e) Imaginary part of IS

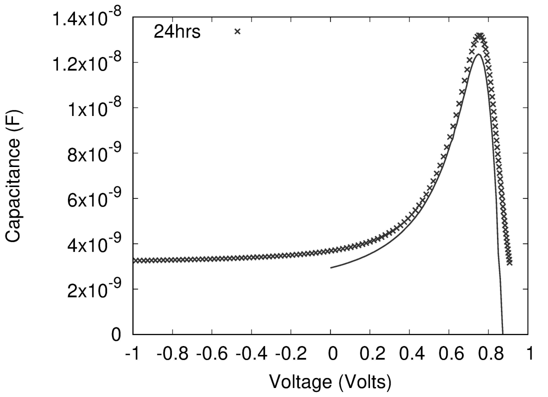

(f) Capacitance–Voltage

Supplementary Figure 17. Fits of the model to the 100 nm thick device aged for 24 h for (a) dark JV, (b) light JV, (c) suns–$V_{\rm oc}$, (d) real part of IS spectra, (e) imaginary part of IS spectra, and (f) capacitance–voltage experimental data.

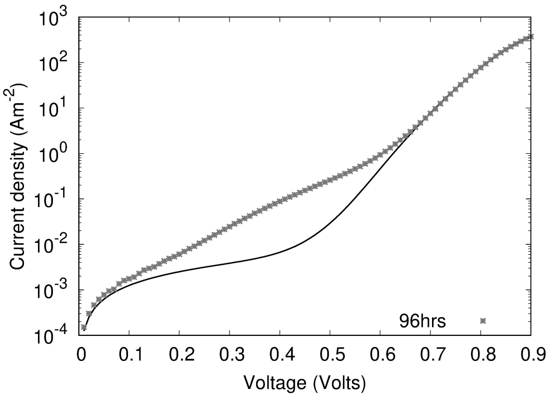

(a) Dark JV

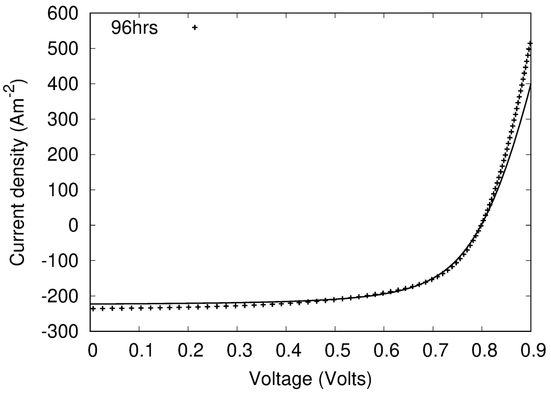

(b) Light JV

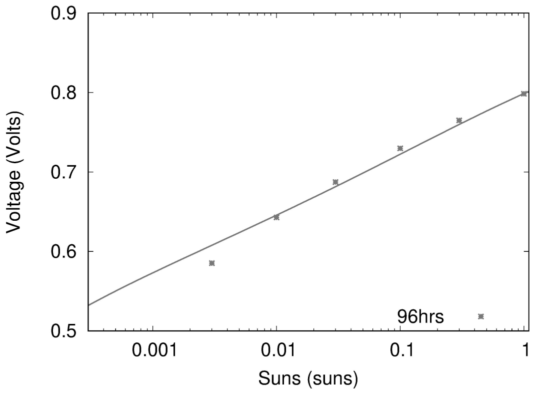

(c) Suns–V_{oc}

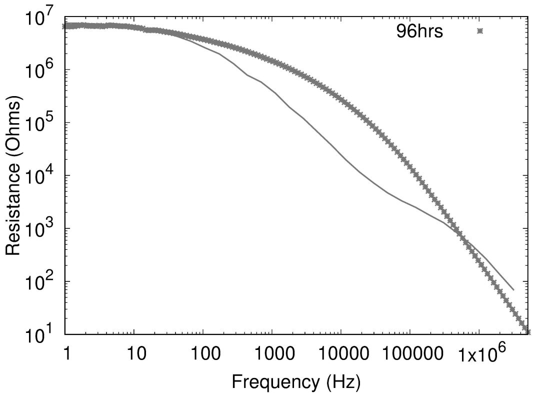

(d) Real part of IS

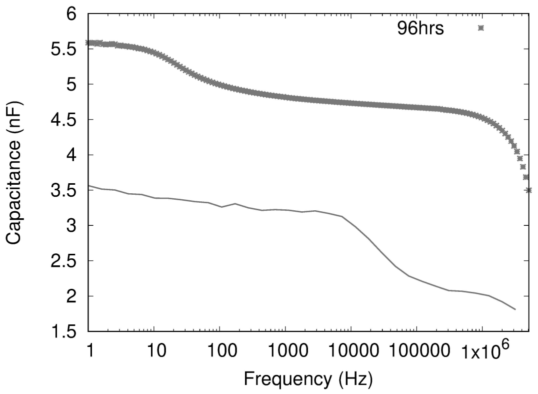

(e) Imaginary part of IS

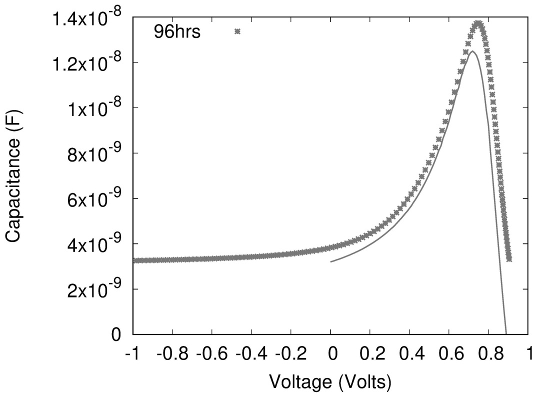

(f) Capacitance–Voltage

Supplementary Figure 18. Fits of the model to the 100 nm thick device aged for 96 h for (a) dark JV, (b) light JV, (c) suns–V_{oc}, (d) real part of IS spectra, (e) imaginary part of IS spectra, and (f) capacitance–voltage experimental data.

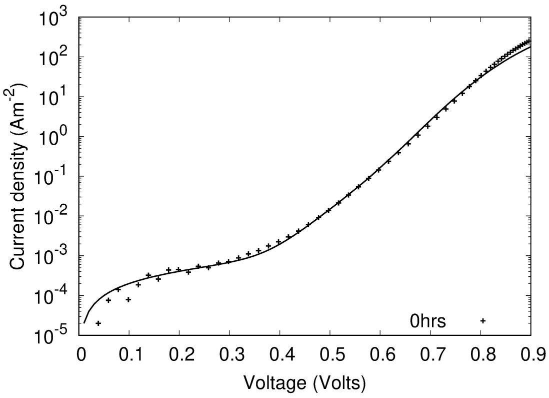

(a) Dark JV

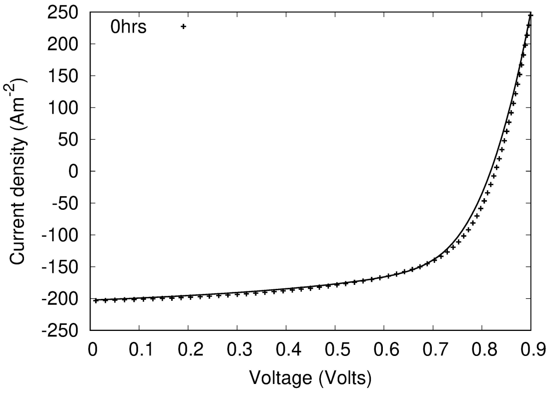

(b) Light JV

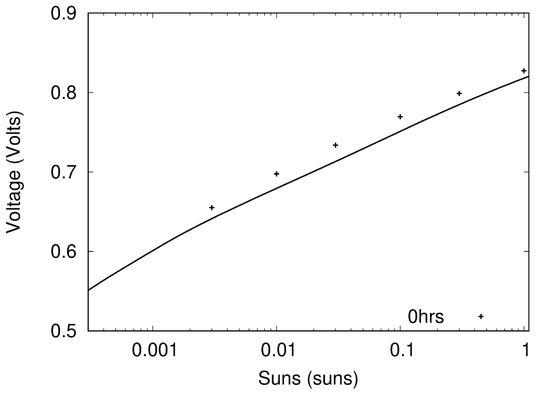

(c) Suns–V_{oc}

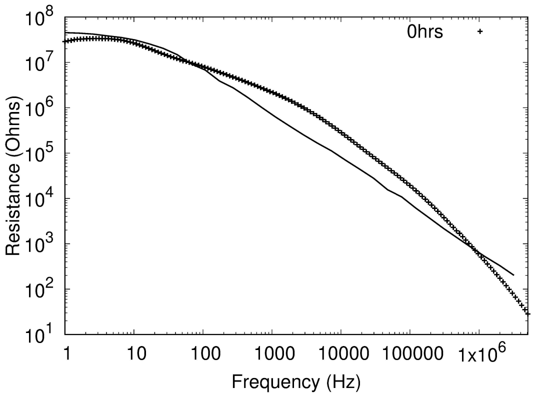

(d) Real part of IS

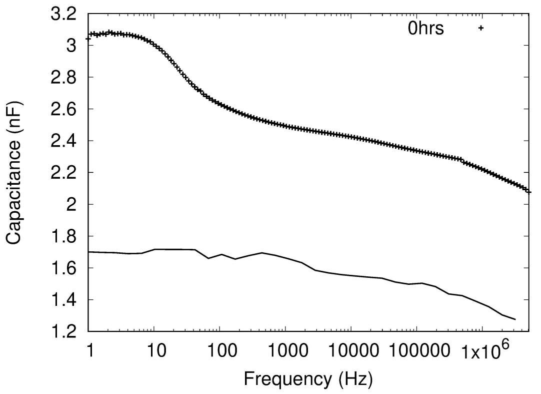

(e) Imaginary part of IS

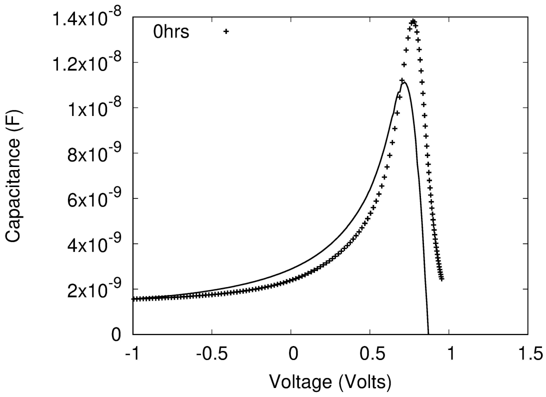

(f) Capacitance–Voltage

Supplementary Figure 19. Fits of the model to the 200 nm thick fresh device for (a) dark JV, (b) light JV, (c) suns–V_{oc}, (d) real part of IS spectra, (e) imaginary part of IS spectra, and (f) capacitance–voltage experimental data.

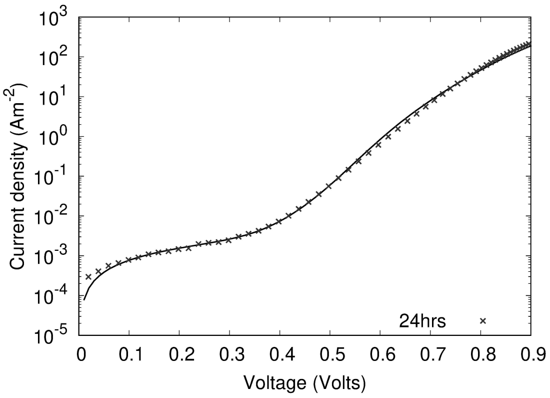

(a) Dark JV

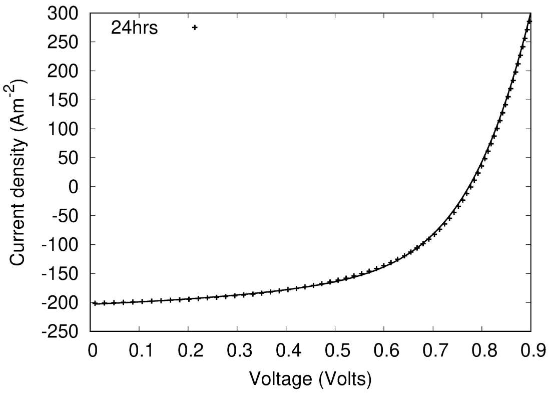

(b) Light JV

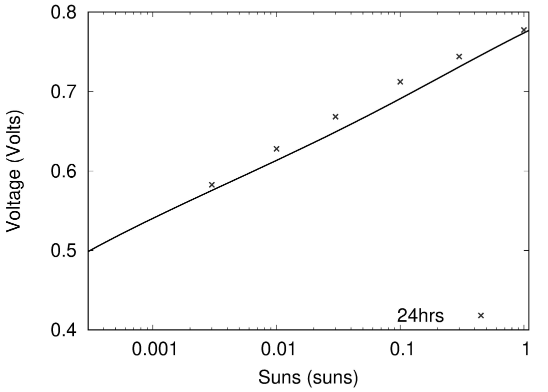

(c) Suns–V_{oc}

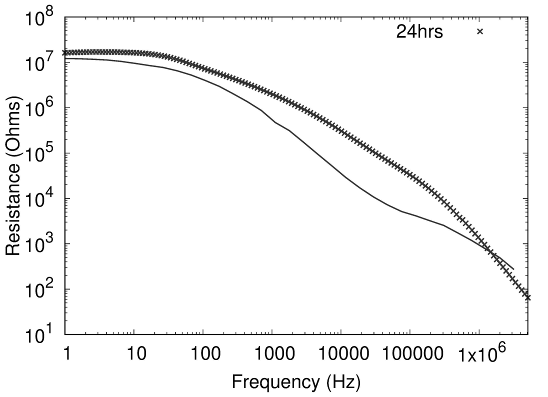

(d) Real part of IS

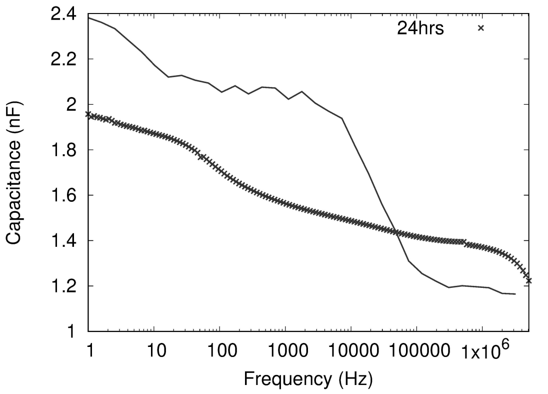

(e) Imaginary part of IS

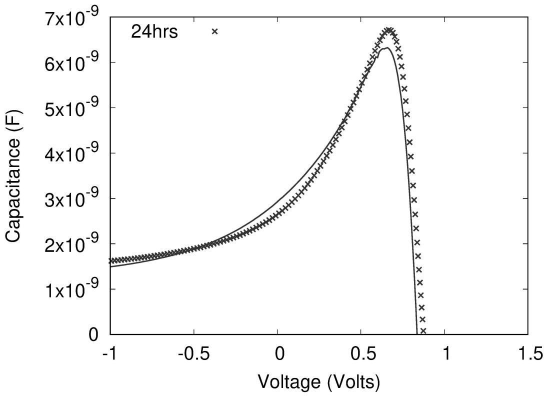

(f) Capacitance–Voltage

Supplementary Figure 20. Fits of the model to the 200 nm thick device aged for 24 h for (a) dark JV, (b) light JV, (c) suns–V_{oc}, (d) real part of IS spectra, (e) imaginary part of IS spectra, and (f) capacitance–voltage experimental data.

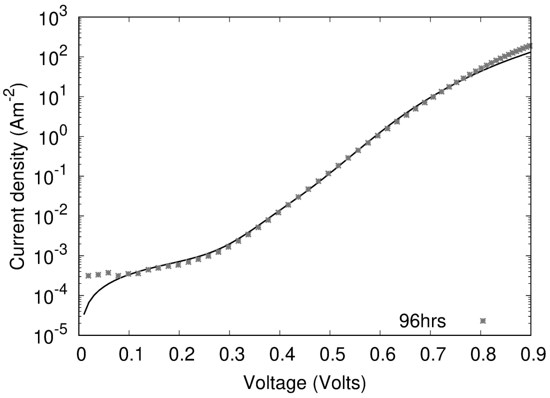

(a) Dark JV

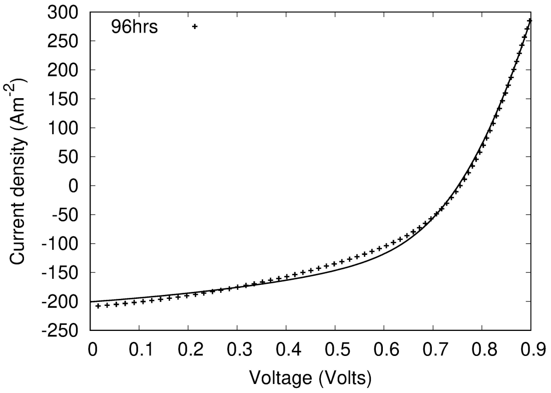

(b) Light JV

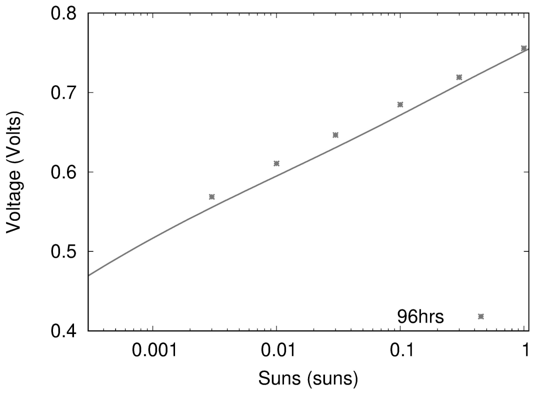

(c) Suns–$V_{\rm oc}$

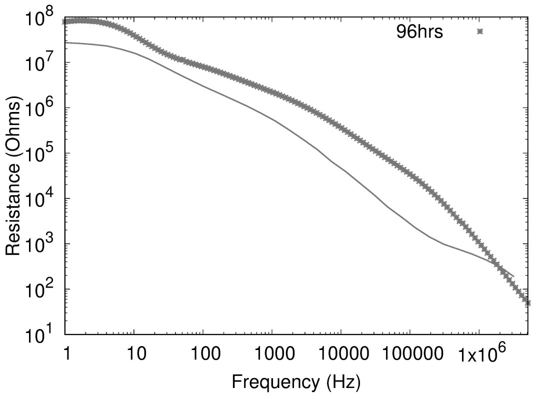

(d) Real part of IS

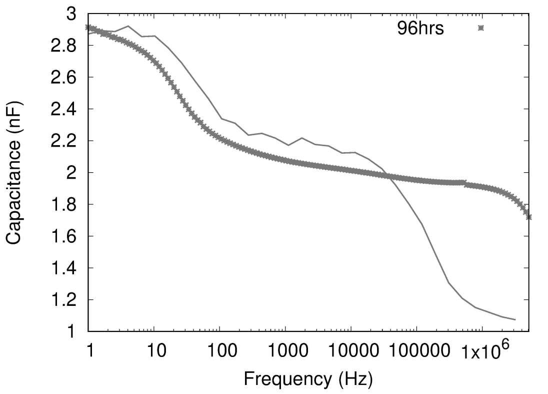

(e) Imaginary part of IS

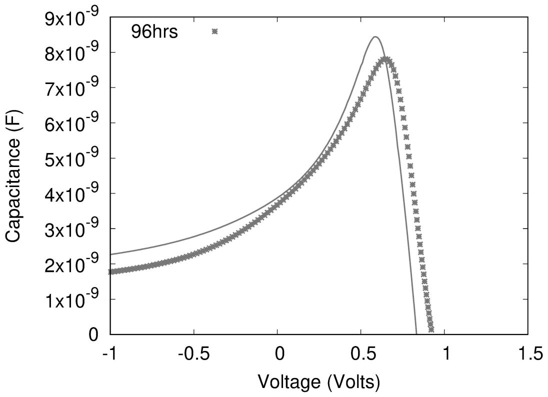

(f) Capacitance–Voltage

Supplementary Figure 21. Fits of the model to the 200 nm thick device aged for 96 h for (a) dark JV, (b) light JV, (c) suns–$V_{\rm oc}$, (d) real part of IS spectra, (e) imaginary part of IS spectra, and (f) capacitance–voltage experimental data.

Supplementary References

[1] M. Unser, A. Aldroubi, and M. Eden, IEEE Transactions on Signal Processing **41**, 821 (1993).
[2] L. Lajaunie, F. Boucher, R. Dessapt, and P. Moreau, Phys. Rev. B **88**, 115141 (2013).
[3] L. Perdigón-Toro, L. Q. Phuong, S. Zeiske, K. Vandewal, A. Armin, S. Shoaee, and D. Neher, ACS Energy Lett. **6**, 557 (2021).
[4] J. Vollbrecht and V. V. Brus, Adv. Electron. Mater. **6**, 2000517 (2020).
[5] A. Foertig, J. Rauh, V. Dyakonov, and C. Deibel, Phys. Rev. B **86**, 115302 (2012).
[6] S. Schiefer, B. Zimmermann, and U. Würfel, J. Appl. Phys. **115**, 044506 (2014).
[7] S. Laux, IEEE Trans. Comput.-Aided Des. Integr. Circuits Syst. **4**, 472 (1985).
