# Supplementary material for: Traps and transport resistance are the next frontiers for stable non-fullerene acceptor solar cells
Source: Nat Commun. 2022 Jul 1;13:3786. doi: 10.1038/s41467-022-31326-z (PMC9249898; doi:10.1038/s41467-022-31326-z)
Supplement: Supplementary file 2 — Solar Cells Reporting Summary [file 41467_2022_31326_MOESM2_ESM.pdf]

nature research

Corresponding author(s): Carsten Deibel (deibel@physik.tu-chemnitz.de)

Solar Cells Reporting Summary

Nature Research wishes to improve the reproducibility of the work that we publish. This form is intended for publication with all accepted papers reporting the characterization of photovoltaic devices and provides structure for consistency and transparency in reporting. Some list items might not apply to an individual manuscript, but all fields must be completed for clarity.

For further information on Nature Research policies, including our data availability policy, see Authors & Referees.

Experimental design

Please check: are the following details reported in the manuscript?

1. Dimensions

Item	Yes/No	Details
Area of the tested solar cells	☒ Yes ☐ No	Methods -> Solar cell devices
Method used to determine the device area	☒ Yes ☐ No	Methods -> Solar cell devices

2. Current-voltage characterization

Item	Yes/No	Details
Current density-voltage (J-V) plots in both forward and backward direction	☒ Yes ☐ No	Methods -> Current--voltage characterization
Voltage scan conditions<br>*For instance: scan direction, speed, dwell times*	☒ Yes ☐ No	Methods -> Current--voltage characterization
Test environment<br>*For instance: characterization temperature, in air or in glove box*	☒ Yes ☐ No	Methods, Results
Protocol for preconditioning of the device before its characterization	☒ Yes ☐ No	Methods -> Aging
Stability of the J-V characteristic<br>*Verified with time evolution of the maximum power point or with the photocurrent at maximum power point; see ref. 7 for details.*	☐ Yes ☒ No	not included in our measurements because the stability of the maximum power point under prolonged illumination and/or bias voltage is not related to the claims of this study

3. Hysteresis or any other unusual behaviour

Item	Yes/No	Details
Description of the unusual behaviour observed during the characterization	☐ Yes ☒ No	no unusual behavior found
Related experimental data	☐ Yes ☒ No	no unusual behavior found

4. Efficiency

Item	Yes/No	Details
External quantum efficiency (EQE) or incident photons to current efficiency (IPCE)	☒ Yes ☐ No	Supporting Note III, Figure 2
A comparison between the integrated response under the standard reference spectrum and the response measure under the simulator	☐ Yes ☒ No	class A solar simulator, additional EQE each time would have delayed further measurements a lot
For tandem solar cells, the bias illumination and bias voltage used for each subcell	☐ Yes ☒ No	no tandem solar cells

5. Calibration

Light source and reference cell or sensor used for the characterization

☐ Yes — *State where this information can be found in the text.*
☒ No — class A solar simulator; regular maintenance in May 2022 showed spectrum still within class A standard

Confirmation that the reference cell was calibrated and certified

☐ Yes — *State where this information can be found in the text.*
☒ No — no reference cell used

Calculation of spectral mismatch between the reference cell and the devices under test

☐ Yes — *State where this information can be found in the text.*
☒ No — no reference cell used

6. Mask/aperture

Size of the mask/aperture used during testing

☐ Yes — *State where this information can be found in the text.*
☒ No — no aperture; no sample positioning system in operation to ensure proper mask alignment

Variation of the measured short-circuit current density with the mask/aperture area

☐ Yes — *State where this information can be found in the text.*
☒ No — no apertures used

7. Performance certification

Identity of the independent certification laboratory that confirmed the photovoltaic performance

☐ Yes — *State where this information can be found in the text.*
☒ No — no certification due to us not aiming for maximum performance

A copy of any certificate(s)
Provide in Supplementary Information

☐ Yes — *State where this information can be found in the text.*
☒ No — no certificates available

8. Statistics

Number of solar cells tested

☐ Yes — *State where this information can be found in the text.*
☒ No — one substrate per aging condition, one sample per substrate measured

Statistical analysis of the device performance

☐ Yes — *State where this information can be found in the text.*
☒ No — not of interest for our mechanism-oriented evaluations

9. Long-term stability analysis

Type of analysis, bias conditions and environmental conditions
For instance: illumination type, temperature, atmosphere humidity, encapsulation method, preconditioning temperature

☒ Yes — Methods -> Aging, Results
☐ No — *Explain why this information is not reported/not relevant.*
